# Safety Evaluation of Soy Leghemoglobin Protein Preparation Derived From *Pichia pastoris*, Intended for Use as a Flavor Catalyst in Plant-Based Meat

**DOI:** 10.1177/1091581818766318

**Published:** 2018-04-11

**Authors:** Rachel Z. Fraser, Mithila Shitut, Puja Agrawal, Odete Mendes, Sue Klapholz

**Affiliations:** 1 Impossible Foods Inc., Redwood City, CA, USA; 2 Product Safety Labs, Dayton, NJ, USA

**Keywords:** leghemoglobin, *Pichia pastoris*, flavor, toxicology, safety

## Abstract

The leghemoglobin protein (LegH) from soy (*Glycine max*) expressed in *Pichia pastoris* (LegH preparation, LegH Prep) imparts a meat-like flavor profile onto plant-based food products. The safety of LegH Prep was evaluated through a series of in vitro and in vivo tests. The genotoxic potential of LegH Prep was assessed using the bacterial reverse mutation assay (Ames test) and the in vitro chromosome aberration test. LegH Prep was nonmutagenic and nonclastogenic in each test, respectively. Systemic toxicity was assessed in a 28-day dietary study in male and female Sprague Dawley rats. There were no mortalities associated with the administration of LegH Prep. There were no clinical observations, body weight, ophthalmological, clinical pathology, or histopathological changes attributable to LegH Prep administration. There were no observed effects on male reproduction in this study, but the suggestion of a potential estrous cycle distribution effect in female rats prompted a second comprehensive 28-day dietary study in female Sprague Dawley rats. This study demonstrated that female reproductive parameters were comparable between rats treated with LegH Prep and concurrent control rats. These studies establish a no observed adverse effect level of 750 mg/kg/d LegH, which is over 100 times greater than the 90th percentile estimated daily intake. Collectively, the results of the studies presented raise no issues of toxicological concern with regard to LegH Prep under the conditions tested.

## Introduction

Western diets containing meat have a larger negative impact on the environment compared to more sustainable plant-based diets.^1–4^ However, due to both social and personal reasons, many consumers are reluctant to reduce the amount of meat they eat.^2,5,6^ To date, plant-based diets have been limited to small populations, such as consumers who follow vegetarian or vegan principles.^7–9^ One potential way to catalyze widespread shift to more sustainable, plant-based diets is to create meat directly from plants that satisfies the tastes of meat consumers.^7,8,10^ Achieving that goal would require products that recreate the sensory properties that people crave in meat, including texture, mouthfeel, taste, smell, and cooking experience, based on an understanding of the biochemical origins of meat sensory attributes.

An investigation of the molecular mechanisms underlying the unique flavors and aromas of meat led to the discovery that heme is the critical catalyst of the chemical reactions that transform simple biomolecules into the complex array of odorants and flavor molecules that define the characteristic flavor profile of meat.^11^ Heme is an iron-containing porphyrin ring that exists as a protein cofactor in all branches of life and is essential for most biochemical processes involving molecular oxygen.^12^ Myoglobin and hemoglobin proteins from animal meat tissues have been consumed throughout human history and represent an important source of dietary iron.^13,14^ Plants contain symbiotic and nonsymbiotic hemoglobin proteins, both of which share a common ancestor with animal hemoglobins.^15^ Symbiotic plant hemoglobins, also known as leghemoglobins, are present in the root nodules of leguminous plants.^16^ Leghemoglobin controls the oxygen concentration in the area surrounding symbiotic nitrogen-fixing bacteria.^15–17^ Nonsymbiotic plant hemoglobins are expressed in the stems, roots, cotyledon, and leaves and are involved in oxygen homeostasis pathways.^18,19^ Due to the presence of nonsymbiotic hemoglobins in legumes, cereals, and other plants,^12,15,20–22^ low levels of plant heme proteins are widely consumed in the human diet.^23–25^

In animal-derived meat, upon cooking, myoglobin, a heme protein exceptionally abundant in animal muscle tissue, unfolds and exposes the heme cofactor. The cofactor then catalyzes a series of reactions that transform the amino acids, nucleotides, vitamins, and sugars naturally found in animal muscle tissue, into a highly specific and diverse set of hundreds of flavor and aroma compounds, the combination of which create the unmistakable and distinctive flavor profile of meat. In plant-based meats, the leghemoglobin protein (LegH) from soy (*Glycine max*), a close structural ortholog of myoglobin, performs a crucial, parallel role: It unfolds upon cooking, releasing its heme cofactor to catalyze reactions that can transform the same ubiquitous biomolecules, isolated from plant-based sources, into the array of compounds that comprise the unique flavor and aroma of meat.^11^ Although the primary amino acid sequence of LegH is highly divergent from the sequence of animal hemoglobins and myoglobins, the 3-dimensional structure is highly similar.^26^ Additionally, the heme cofactor bound to LegH (heme B) is identical to heme found in animal meat, which has a long history of safe use in the human diet.^14^ The iron from LegH has an equivalent bioavailability to iron from bovine hemoglobin when supplemented in a food matrix.^27^ However, because soy root nodules, and thus LegH, are not commonly consumed in the human diet, the safety properties of LegH remain not fully understood.

In pursuit of the intended use in ground beef analogue products, the gene encoding a soy LegH was introduced into the genome of the yeast *Pichia pastoris*, enabling the production of high levels of soy LegH preparation (LegH Prep). The total protein fraction of LegH Prep contains at least 65% LegH. The remaining proteins are from the Pichia host. *Pichia pastoris* is nontoxigenic and nonpathogenic and has been used in the recombinant expression of both Generally Recognized as Safe (GRAS) and US Food and Drug Administration (FDA)-approved proteins.^28,29^ Previously, the LegH and Pichia proteins within LegH Prep were evaluated for potential risk of allergenicity and toxicity in accordance with the CODEX Alimenterius Commission 2003/2009 guidelines for genetically modified foods and for novel food ingredients.^30^ The analysis included literature search, sequence homology comparison to known allergens and toxins, and sensitivity to pepsin digestion in a simulated gastric fluid. There was no evidence in the scientific literature to suggest allergenicity or toxicity for these proteins, and the LegH protein sequence did not contain significant homology to any known allergens or toxins. Although some of the proteins from the Pichia host present within LegH Prep had modest identity matches to minor airway allergens and/or proteins from toxic organisms, these proteins have analogous identity matches to proteins from the widely consumed yeast *Saccharomyces cerevisiae*, which has no evidence of food allergy or toxicity. Leghemoglobin protein and the Pichia proteins within LegH Prep were readily digested in simulated gastric fluid. Collectively, the authors of this study concluded that there was no evidence to suggest that food products containing LegH Prep posed any significant risk of dietary allergy or toxicity to consumers.^30^

In this study, we evaluated the safety of LegH Prep with both in vitro and in vivo models. To evaluate potential genotoxicity of LegH Prep, a bacterial reverse mutation test (Ames test) and an in vitro chromosome aberration test were performed. These in vitro models showed that LegH Prep is neither mutagenic nor clastogenic. Systemic toxicity was evaluated by a 28-day feeding study in Sprague Dawley rats. An additional 28-day feeding study was performed to evaluate the female estrous cycle and reproductive health. These in vivo models demonstrated no adverse effects attributable to the consumption of LegH Prep at the maximum dose tested, which was more than 100 times greater than the 90th percentile estimated daily intake (EDI) in ground beef analogue products. Collectively, the results of the studies presented raise no issues of toxicological concern with regard to LegH Prep under the conditions tested.

## Materials and Methods

### Test Substance Production and Analysis

The *P pastoris* LegH production strain MXY0291 was derived from the well-characterized parent strain NRRL Y-11430.^31^ MXY0291 was modified to overexpress the gene encoding the soy LegH as well as all 8 enzymes in the native Pichia heme biosynthesis pathway (aminolevulinic acid (ALA) synthase, ALA dehydratase, porphobilinogen deaminase, UPG III synthase, uroporphyrinogen (UPG) III decarboxylase, coproporphyrinogen oxidase, protoporphyrinogen oxidase, and ferrochelatase) using the Pichia *alcohol oxidase 1* promoter (*pAOX1*). MXY0291 was also modified to overexpress the Mxr1 transcriptional activator using the *pAOX1* promoter. The Mxr1 protein activates the *pAOX1* promoter leading to increased expression of *pAOX1*-driven LegH, heme biosynthesis genes, and Mxr1 itself.

Leghemoglobin protein was recombinantly expressed in *P pastoris* MXY0291 during submerged fed-batch fermentation and isolated using filtration-based recovery with food- or pharmaceutical-grade materials. The Pichia production strain (MXY0291) is derived from a nontoxigenic and nonpathogenic, well-characterized strain lineage that has a history of safe use in manufacturing proteins for use in food and pharmaceuticals.^28,29,32^ This process is compliant with the Enzyme Technical Association’s guidelines for fermentation-produced microbiologically derived proteins and follows current Good Manufacturing Practices.^33,34^ Soy LegH is expressed during submerged fed-batch fermentation. The *P pastoris* cells in the fermentation broth are lysed by bead mill mechanical shearing. Insoluble material within the lysate is removed by centrifugation and microfiltration. Ultrafiltration is used to concentrate the soy LegH. The resulting concentrated liquid is formulated with sodium chloride and sodium ascorbate and stored as a frozen liquid. Leghemoglobin protein preparation may be stored at −20°C as a frozen liquid for at least 12 months with no observable change in soy leghemoglobin stability or performance in ground beef analogue products. Leghemoglobin protein preparation is a frozen liquid, and the entire final preparation was used for all in vitro safety tests performed in this study. Leghemoglobin protein preparation specifications and batch analysis for the lots used for safety testing are presented in Supplemental Table S1. To aid with homogeneous mixing into the animal diet, LegH Prep was freeze-dried prior to use for all in vivo studies.

During each of the in vivo studies described below, the LegH concentrations within the neat test substance and animal feed samples were analyzed by Impossible Foods using high-performance liquid chromatography (HPLC) to evaluate test substance concentration, stability, and homogeneity. Leghemoglobin protein preparation was extracted from the feed by adding 50 mmol/L potassium phosphate pH 7.4, 150 mmol/L sodium chloride to each feed sample followed by 1 hour of end-over-end rotation. High-performance liquid chromatography was performed using an Agilent 1100 Series instrument with an ACQUITY xBridge BEH125 SEC 7.8 × 150 mm ID 3.5 μm column (Waters, Milford, MA). Leghemoglobin protein concentration was determined by integration of the 415 nm absorbance at the LegH retention time.

### Estimated Daily Intake

Ground beef analogue products will be formulated to contain approximately the same amount of heme as beef. This equates to a typical and maximum usage rate of 0.6% and 0.8% LegH, respectively. The EDI for LegH within ground beef analogue products was calculated based on 100% capture of the US ground beef market, which is approximately 500 times higher than the market size for all meat and poultry analogue products (note 1). The national mean daily consumption of ground beef for males and females aged 2 and older is 25 g/day (59 g beef/person/d × 42% of beef sales are ground beef).^35,36^ Replacement of ground beef with ground beef analogue on a one-for-one basis would result in typical (0.6% LegH use rate) and maximum (0.8% LegH use rate) Leghemoglobin protein EDIs of 150 and 200 mg/person/d, respectively. In accordance with FDA guidelines, the 90th percentile EDI was calculated as 2 times the maximum EDI or 400 mg/person/d LegH, which corresponds to 6.67 mg/kg bodyweight/day assuming an average body weight of 60 kg. The 90th percentile was used as a basis for safety testing.

### Bacterial Reverse Mutation Assay (Ames Test)

The Ames test (reverse mutation test) was performed by Product Safety Labs (Product Safety Labs (PSL); Dayton, New Jersey) and was conducted in accordance with US FDA good laboratory practice (GLP) regulations (21 CFR Part 58). The assay design was based on The Organization for Economic Co-operation and Development (OECD) guideline 471^37^ and ICH Guidelines S2A and S2B.^38^ Five bacterial strains were evaluated (*Salmonella typhimurium* (ST) TA98, TA100, TA1535, and TA1537 and *Escherichia coli* (EC) WP2uvrA; Molecular Toxicology, Inc, Boone, North Carolina) according to the plate incorporation and preincubation methods both in the presence and in the absence of a metabolic activation system (S9 mix). Sterile water served as the negative control and as the vehicle, while 5 mutagens including sodium azide (NaN_3_), ICR 191 acridine, daunomycin, methyl methanesulfonate, and 2-aminoanthracene (2-AA; Molecular Toxicology, Inc, Boone, North Carolina) were used as the positive controls. Water was also used as the solvent for the positive controls except for 2-AA, which was prepared in dimethyl sulfoxide. The initial test followed the plate incorporation method in which the following materials were mixed and poured over the surface of a minimal agar plate: 100 μL of the prepared test solutions, negative (vehicle) control, or prepared positive control substance; 500 μL S9 mix or substitution buffer; 100 μL bacteria suspension (ST or EC); and 2000 μL overlay agar maintained at approximately 45°C.

Plates were prepared in triplicate and uniquely identified. Appropriate sterility control check plates (treated with critical components in the absence of bacteria) were included as a standard procedural check. After pouring, the plates were placed on a level surface until the agar gelled, then inverted, and incubated at 37°C ± 2°C until growth was adequate for enumeration (approximately 65 ± 3 hours).

The confirmatory test employed the preincubation modification of the plate incorporation test. The test or control substances, bacteria suspension, and S9 (or substitution buffer) were incubated under agitation for approximately 30 minutes at approximately 37°C ± 2°C prior to mixing with the overlay agar and pouring onto the minimal agar plates before proceeding as described for the initial test. Following incubation, the revertant colonies were counted manually and/or with the aid of a plate counter (Colony Plate Reader: Model Colony-Doc-It, Colony Doc-itTM 125 Imaging station UVP, P/N 97-0539-01). To be considered valid, the background lawn for vehicle control plates had to appear slightly hazy with abundant microscopic nonrevertant bacterial colonies. The mean revertant colony counts for each strain treated with the vehicle had to lie close to or within the expected range, taking into account the laboratory historical control range. For each experimental point, the mutation factor (MF) was calculated by dividing the mean revertant colony count by the mean revertant colony count for the corresponding concurrent vehicle control group. The results were considered to be positive when the MF was increased at least by a factor of 2 for strains TA98, TA100, and WP2 uvrA or by at least a factor of 3 for strains TA1535 and TA1537. In addition, any increases had to be dose related and/or reproducible, that is, increases must be obtained at more than 1 experimental point (at least 1 strain, more than 1 dose level, and more than 1 occasion or with different methodologies).

### Chromosome Aberration Assay in Human Peripheral Blood Lymphocytes

The chromosomal aberration assay was conducted at Eurofins Biopharma (Munich, Germany) in compliance with the German GLP regulations according to 19b Abs. 1 chemikaliengesetz^39^ and the protocol procedures described in the Term Tests for Genetic Toxicity and OECD 473, In Vitro Mammalian Chromosome Aberration Test^40,41^ and the European Commission Regulation (EC) no440/2008 B.10.^42^ The study was conducted using human peripheral blood (BLD) lymphocytes (HPBL) in both the absence and the presence of the chemically induced rat liver S9 metabolic activation system (Trinova Biochem, Giessen, Germany). Peripheral BLD lymphocytes were obtained from healthy nonsmoking donors who had no recent history of exposure to genotoxic chemicals and radiation. Peripheral BLD lymphocytes were cultured in complete medium (Roswell Park Memorial Institute (RPMI) 1640 containing 15% heat inactivated fetal bovine serum, 0.24 g/mL of phytohemagglutinin, 100 units penicillin, and 100 µg/mL streptomycin). The cultures were incubated under standard conditions (37°C in a humidified atmosphere of 5% CO_2_ in air) for 48 hours. The cells were treated for periods of 4 or 24 hours in the nonactivated test system and for a period of 4 hours in the S9-activated test system. All cells were harvested 24 hours after treatment initiation. Cyclophosphamide and ethylmethanesulfonate (Sigma-Aldrich, Missouri) were evaluated as the concurrent positive controls for treatments with and without S9, respectively.

In addition to the mitotic index determination, the proliferation index of selected samples (negative control and high doses of LegH Prep) was calculated using the BrdU (5-bromo-2’-deoxyurindine) technique. The proliferation index was calculated using Equation 1 (where M1 is the first generation, M2 is the second generation, and M3 is the third generation) based on the number of cell divisions undertaken during the experiment.

1 $$\text{PI}=\;\frac{1\left(\text{\% cells in M}1\right)+2\left(\text{\% cells in M}2\right)+\left(\text{\% cells in M}3\right)}{100}$$

### Fourteen-Day Dietary Palatability and Range Finding Study in Rats

This study was conducted at PSL following the OECD 407 Guidelines for Testing of Chemicals^43^ and Food Ingredients and US FDA Toxicological Principles for the Safety Assessment of Food Ingredients^21^ IV.C.4.a^44^ and was approved by the Institutional Animal Care and Use Committees (IACUC) of PSL. PSL is Association for Assessment and Accreditation of Laboratory Animal Care accredited and certified in the appropriate care of all live experimental animals and maintains current staff training ensuring animals were handled humanely during the experimental phase of this study in compliance with the National Research Council’s 2011 Guide for the Care and Use of Laboratory Animals (8th ed.).^45^ Charles River Laboratories (CRL) Sprague-Dawley CD International Genetic Standardization (IGS) rats were purchased from Charles River Laboratories (Kingston, New York) and subsequently quarantined and acclimated to the PSL facilities. Animals were maintained in a temperature- and humidity-controlled room at 19°C to 22°C and 41% to 65%, respectively, under a 12-hour light–dark cycle and fed a standard Envigo Teklad Global 16% Protein Rodent Diet #2016 (Envigo Laboratories, Inc, Indianapolis, Indiana). The diet and filtered tap water were supplied ad libitum. All contaminants within the diet and filtered tap water were within acceptable regulatory standards. The animals were housed in pairs and received enrichment activities such as chew sticks throughout the duration of the study. Forty-eight animals were selected for the test (7-8 weeks of age at dosing; weighing 230-264 g [males] and 158-181 g [females]) and distributed into 4 groups with 6 males and 6 females each (1 control group per sex and 3 dietary levels per sex). The freeze-dried LegH Prep was administered in the diet at concentrations that targeted 0 (group 1, control), 125 (group 2, low), 250 (group 3, medium), and 500 (group 4, high) mg/kg/d of the soy leghemoglobin-active ingredient (Supplemental Table S10). Food consumption was determined by biweekly feed jar weight as well as weighing any feed that was spilled into the cage. For food consumption measurements, consumed feed weight was divided by 2 to account for paired housing.

The animals were observed for viability, signs of gross toxicity, and behavioral changes at least once daily during the study and weekly for a battery of detailed observations. Body weights were recorded 2 times during the acclimation period (including prior to dosing on study day 1) and on study days 3, 7, 10, and 14. Individual food consumption, based on total food consumed per cage divided by 2 to account for paired housing, was also recorded to coincide with body weight measurements. Food efficiency was calculated by dividing the mean daily body weight gain by the mean daily food consumption. The animals were fasted overnight prior to BLD collection. Samples were collected from all animals for hematology evaluation via the inferior vena cava under isoflurane anesthesia during the necropsy procedure. Approximately 500 μL of BLD were collected in a precalibrated tube containing dipotassium ethylenediaminetetraacetic acid (K_2_EDTA). All clinical pathology samples were sent to DuPont Haskell Global Centers for Health and Environmental Sciences (Newark, Delaware) for analysis. Clinical pathology included the following hematology analyses: erythrocyte count (RBC), hematocrit (HCT), mean corpuscular hemoglobin (MCH), absolute reticulocyte count (ARET), total white BLD cell (WBC), and differential leukocyte count, MCH concentration (MCHC), hemoglobin concentration (HGB), mean corpuscular volume (MCV), red cell distribution width (RDW), and platelet count (PLT). Gross necropsy was performed on study day 15, and the animals were evaluated for any macroscopic changes. Histological examination was performed on the liver, spleen, and bone marrow of the animals from the vehicle control and high dose (groups 1 and 4, respectively). Slide preparation was performed by Histo-Scientific Research Laboratories (HSRL, Mount Jackson, Virginia), and histopathological assessment was performed by a board-certified veterinary pathologist at PSL.

### Twenty-Eight-Day Dietary Feeding Study in Rats

This 28-day feeding study was conducted at PSL in accordance with GLP and follows OECD Guidelines for Testing of Chemicals, Section 4 Health Effects (Part 408): Repeated Dose 90-day Oral Toxicity Study in rodents^40,46^ and the US FDA Toxicological Principles for the Safety Assessment of Food Ingredients, IV.C.4.a.^44^ and was approved by the IACUC of PSL. Adult CRL Sprague-Dawley CD IGS rats were purchased from Charles River Laboratories and subsequently quarantined and acclimated to the PSL facilities as described above. Eighty rats were selected for testing, using acceptance criteria described above, and distributed into 4 groups with 10 males and 10 females per group (1 control group per sex and 3 dietary dose levels per sex). The freeze-dried LegH Prep was administered in the diet at concentrations that targeted 0 (group 1, control), 250 (group 2, low), 500 (group 3, medium), and 750 (group 4, high) mg/kg/d of the soy leghemoglobin active ingredient (Supplemental Table S11). Food consumption was determined as described above.

Prior to study initiation and again on study day 23, the eyes of all rats were examined by focal illumination and indirect ophthalmoscopy. Clinical observations, food consumption, body weight, and food efficiency were evaluated as described above. On study day 22, samples were collected from all animals for hematology, serum chemistry, and urinalysis evaluation. Blood samples for hematology and serum chemistry were collected via sublingual bleeding under isoflurane anesthesia. Approximately 500 μL of BLD were collected in a precalibrated tube containing K_2_EDTA for hematology assessments. The whole BLD samples were stored under refrigeration and shipped on cold packs. Approximately 1000 μL of BLD were collected into a tube containing no preservative for serum chemistry assessments. Hematology included RBC, HCT, MCH, ARET, total WBC, and differential leukocyte count, MCHC, HGB, MCV, RDW, and PLT. Coagulation included prothrombin time and activated partial thromboplastin time (APTT). Clinical chemistry included serum aspartate amino transferase, sorbitol dehydrogenase, total bilirubin, BLD creatinine, triglycerides, total serum protein, globulin (GLOB), inorganic phosphorus, potassium (K), serum alanine aminotransferase, alkaline phosphatase, blood urea nitrogen, total cholesterol, fasting glucose (GLUC), albumin (ALB), calcium (CALC), sodium (Na), and chloride (Cl). Urinalysis included, quality, color (COL), clarity, urine volume (UVOL), microscopic urine sediment examination, pH, GLUC, specific gravity, protein (UMTP), ketone, bilirubin, blood (BLD), and urobilinogen. At terminal killing, all animals were euthanized by exsanguination from the abdominal aorta under isoflurane anesthesia, and BLD was collected for evaluation of coagulation parameters. All clinical pathology samples were sent to DuPont Haskell Global Centers for Health and Environmental Sciences for analysis. All animals in the study were subjected to a full necropsy, which included examination of the external surface of the body, all orifices, and the thoracic, abdominal, and cranial cavities and their contents. Tissues/organs representing systems were collected and preserved in 10% neutral-buffered formalin with the exception of the eyes, testes, and epididymides, which were preserved in Davidson’s fixative before transfer to ethanol. A subset of tissues/organs were weighed wet as soon as possible after dissection to avoid drying including adrenal glands, kidneys, spleen, brain, liver, thymus, testes, epididymides, ovaries with oviducts, uterus, and heart. The fixed tissues were trimmed, processed, embedded in paraffin, sectioned with a microtome, placed on glass microscope slides, stained with hematoxylin and eosin, and examined by light microscopy. Histological examination was performed on the complete set of preserved organs and tissues of the animals from the vehicle control and high-dose groups (groups 1 and 4, respectively) as detailed in the OECD 408 guidelines, with the exception of the female reproductive organs, which were evaluated at all dose levels. Slide preparation and histopathological assessment was performed by a board-certified veterinary pathologist at HSRL. A pathology peer review was performed by a board-certified veterinary pathologist at Regan Path/Tox Services (Ashland, Ohio) for all female reproductive tissues.

### Twenty-Eight-Day Investigative Study With a 14-Day Predosing Estrous Cycle Determination

This study was conducted at PSL, and the protocol was approved by the IACUC of PSL. Adult CRL Sprague-Dawley CD IGS female rats were purchased from Charles River Laboratories and subsequently quarantined and acclimated at the PSL facilities as described above. Sixty rats were selected for testing using the acceptance criteria described above, and animals were distributed into 4 groups with 15 females per group (1 control group and 3 dietary dose levels). Freeze-dried LegH Prep was administered in the diet at concentrations that targeted 0 (group 1, control), 250 (group 2, low), 500 (group 3, medium), and 750 (group 4, high) mg/kg/d of the soy leghemoglobin active ingredient (Supplemental Table S12). Food consumption was determined as described above. The estrous cycle was evaluated daily by vaginal cytology for a period of 14 days prior to administration of the test substance and for the last 2 weeks of the 28-day period of test substance administration. Estrous cycle stage was not evaluated for the first 2 weeks of the dosing period to avoid overmanipulating the animals. For each 14-day period, average estrous cycle length was calculated for each animal and subsequently each group. Clinical observations, food consumption, body weight, and food efficiency were evaluated as described above. All animals were subjected to a full necropsy, which included examination of the external surface of the body, all orifices, and the thoracic, abdominal, and cranial cavities and their contents. Female reproductive organs were collected and preserved in 10% neutral buffered formalin. The ovaries with oviducts and uterus were weighed wet as soon as possible after dissection to avoid drying. Reproductive tissues were fixed and examined as described above. Estrous cycle stage was recorded for all animals. Histological examination was performed on the preserved organs and tissues for group 1 and group 4 animals. Slide preparation was performed by Histoserv Inc (Germantown, Maryland), and histopathological assessment was performed by a board certified veterinary pathologist at Regan Path/Tox Services.

### Statistical Analyses

Mean and standard deviations (SDs) were calculated for all quantitative data. For the Chromosome Aberration Study, the Fisher exact test was used to compare the induction of chromosome aberrations in treated cultures and solvent control. Significance was judged at a probability value of *P* < 0.05. Male and female rats were evaluated separately. Body weights, food consumption, UVOL, hematology, BLD chemistry, absolute and relative organ weights, averages, and standard deviations were calculated and analyzed by Bartlett test for homogeneity of variances and normality.^47^ Where Bartlett test indicated homogeneous variances, treated and control groups were compared using a 1-way analysis of variance (ANOVA). When ANOVA was significant, a comparison of the treated groups to control by Dunnett test for multiple comparisons was performed.^48,49^ Where variances were considered significantly different by Bartlett test, groups were compared using a nonparametric method (Kruskal-Wallis non-parametric ANOVA).^50^ When nonparametric ANOVA was significant, comparison of treated groups to control was performed using Dunn test.^51^ Statistical analysis was performed on all quantitative data for in-life and organ weight parameters using Provantis Version 8, Tables and Statistics, Instem LSS, Staffordshire United Kingdom. Clinical pathology was preliminarily tested via Levene test^52^ for homogeneity and via Shapiro-Wilk test^53^ for normalcy followed by ANOVA followed with Dunnett test.^48,49^

## Results

### Bacterial Reverse Mutation Assay (Ames Test)

The objective of this test was to determine the mutagenic potential of LegH Prep using histidine-requiring strains of *S. typhimurium* (TA98, TA100, TA1535, and TA1537) and a tryptophan-requiring strain of *E. coli* (WP2 uvrA). Leghemoglobin protein preparation was evaluated with and without an exogenous metabolic activation (S9 mix) at levels of 23.384, 74, 233.84, 740, 2338.4, 7400, 23,384 and 74,000 μg/plate, which corresponded to 1.58, 5.0, 15.8, 50, 158, 500, 1580, and 5000 μg/plate of the characterizing component, LegH, with the high level being the standard limit for this test (Supplemental Table S2).

The mean revertant colony counts for each strain treated with the vehicle were close to or within the expected range, considering the laboratory historical control range and/or published values (Supplemental Table S3).^54,55^ The positive control substances caused the expected substantial increases in revertant colony counts in both the absence and the presence of S9 in each phase of the test, confirming the sensitivity of the test and the activity of the S9 mix (Table 1, Supplemental Table S3). No signs of precipitation or contamination were noted throughout the study. Therefore, each phase of the test is considered valid.

**Table 1.** Number of Revertant Colonies and Mutation Factors Without/With Metabolic Activation (S9)—Ames Test.^a,b,c,d,e^

Compound	LegH dose^f^ (µg/plate))	TA98				TA100				TA1535				TA1537				EC WP2 uvrA			
		−S9		±S9		−S9		±S9		−S9		±S9		−S9		±S9		−S9		±S9	
		Mean revertants/plate ± SD				Mean revertants plate ± SD				Mean revertants/plate ± SD				Mean revertants/plate ± SD				Mean revertants/plate ± SD			
		Rep 1	Rep 2	Rep 1	Rep 2	Rep 1	Rep 2	Rep 1	Rep 2	Rep 1	Rep 2	Rep 1	Rep 2	Rep 1	Rep 2	Rep 1	Rep 2	Rep 1	Rep 2	Rep 1	Rep 2
Vehicle	0	25 ± 1.5	21 ± 1.5	27 ± 4.7	26 ± 3.8	103 ± 7.8	103 ± 10.3	119 ± 9.6	120 ± 4.5	18 ± 2.1	13 ± 2.1	13 ± 2.5	11 ± 1.5	13 ± 0.6	8 ± 1.0	12 ± 4.9	12 ± 3.2	40 ± 5.5	34 ± 3.8	52 ± 10.4	41 ± 5.6
Mutation factor		1.00	1.00	1.00	1.00	1.00	1.00	1.00	1.00	1.00	1.00	1.00	1.00	1.00	1.00	1.00	1.00	1.00	1.00	1.00	1.00
LegH	1.58	24 ± 1.7	22 ± 1.5	26 ± 2.1	25 ± 1.2	98 ± 9.5	89 ± 4.7	106 ± 8.1	98 ± 6.5	9 ± 2.1	15 ± 2.6	11 ± 3.5	11 ± 3.6	10 ± 2.3	13 ± 4.5	14 ± 1.2	17 ± 2.5	43 ± 8.1	40 ± 7.0	40 ± 3.5	47 ± 7.2
Mutation factor		0.96	1.05	0.96	0.96	0.96	0.86	0.89	0.82	0.5	1.15	0.85	1.00	0.77	1.63	1.17	1.42	1.08	1.18	0.77	1.15
LegH	5.0	20 ± 0.6	21 ± 6.6	23 ± 2.0	29 ± 6.1	91 ± 3.2	86 ± 7.2	109 ± 4.6	106 ± 4.0	12 ± 5.5	11 ± 2.1	14 ± 0.6	10 ± 3.6	11 ± 3.5	17 ± 6.4	14 ± 3.2	15 ± 3.5	34 ± 7.2	28 ± 7.2	38 ± 4.0	48 ± 1.0
Mutation factor		0.80	1.00	0.85	1.12	0.88	0.83	0.92	0.88	0.67	0.85	1.08	0.91	0.85	2.13	1.17	1.25	0.85	0.82	0.73	1.17
LegH	15.8	23 ± 4.0	19 ± 2.6	28 ± 2.6	23 ± 2.0	97 ± 14.4	96 ± 14.6	102 ± 15.1	104 ± 14.0	10 ± 1.7	16 ± 4.2	13 ± 3.1	10 ± 2.6	11 ± 0.6	8 ± 2.1	14 ± 3.5	12 ± 2.6	42 ± 7.6	41 ± 7.0	53 ± 2.6	37 ± 2.6
Mutation factor		0.92	0.90	1.04	0.88	0.94	0.93	0.86	0.87	0.56	1.23	1.00	0.91	0.85	1.00	1.17	1.00	1.05	1.21	1.02	0.90
LegH	50	22 ± 2.1	28 ± 3.5	27 ± 2.0	22 ± 0.6	89 ± 5.1	101 ± 7.0	108 ± 8.7	110 ± 6.1	12 ± 4.0	11 ± 0.6	9 ± 1.0	10 ± 1.2	13 ± 4.0	11 ± 6.2	10 ± 2.9	8 ± 4.4	46 ± 1.5	32 ± 2.3	43 ± 10.1	44 ± 7.6
Mutation factor		0.88	1.33	1.00	0.85	0.86	0.98	0.91	0.92	0.67	0.85	0.69	0.91	1.00	1.38	0.83	0.67	1.15	0.94	0.83	1.07
LegH	158	20 ± 2.5	21 ± 2.9	26 ± 0.6	25 ± 4.0	98 ± 6.7	94 ± 6.6	94 ± 3.1	92 ± 11.1	12 ± 1.7	13 ± 4.7	13 ± 2.3	8 ± 3.6	11 ± 0.6	8 ± 2.6	14 ± 0.6	11 ± 1.5	45 ± 4.4	31 ± 2.9	45 ± 13.3	41 ± 0.6
Mutation factor		0.80	1.00	0.96	0.96	0.96	0.91	0.79	0.77	0.67	1.00	1.00	0.73	0.85	1.00	1.17	0.92	1.13	0.91	0.87	1.00
LegH	500	22 ± 2.5	21 ± 2.0	25 ± 5.0	28 ± 2.3	96 ± 5.5	81 ± 11.4	99 ± 3.5	89 ± 7.5	11 ± 7.0	11 ± 4.0	11 ± 1.0	12 ± 2.6	9 ± 0.6	10 ± 3.6	8 ± 3.2	10 ± 1.0	41 ± 11.6	36 ± 9.0	57 ± 8.3	52 ± 4.0
Mutation factor		0.88	1.00	0.93	1.08	0.93	0.79	0.83	0.74	0.61	0.85	0.85	1.09	0.69	1.25	0.67	0.83	1.03	1.06	1.10	1.27
LegH	1580	26 ± 0.6	22 ± 4.0	19 ± 2.1	26 ± 6.1	97 ± 5.5	95 ± 10.2	107 ± 3.2	98 ± 7.5	15 ± 5.2	7 ± 2.1	10 ± 3.8	10 ± 2.1	7 ± 0.6	12 ± 2.9	10 ± 1.7	14 ± 6.5	46 ± 3.6	32 ± 5.1	47 ± 6.7	47 ± 3.5
Mutation factor		1.04	1.05	0.70	1.00	0.94	0.92	0.90	0.82	0.83	0.54	0.77	0.91	0.54	1.50	0.83	1.17	1.15	0.94	0.90	1.15
LegH	5000	23 ± 3.5	24 ± 4.6	28 ± 4.7	30 ± 5.0	100 ± 7.0	96 ± 10.0	101 ± 9.5	111 ± 6.2	12 ± 1.2	12 ± 5.3	13 ± 3.1	11 ± 2.5	8 ± 1.5	14 ± 1.0	13 ± 3.6	16 ± 2.5	39 ± 8.0	44 ± 2.1	53 ± 5.5	39 ± 11.9
Mutation factor		0.92	1.14	1.04	1.15	0.97	0.93	0.85	0.93	0.67	0.92	1.00	1.00	0.62	1.75	1.08	1.33	0.98	1.29	1.02	0.96
Daumonycin^g^	6	801 ± 19.9	309 ± 9.6	–	–	–	–	–	–	–	–	–	–	–	–	–	–	–	–	–	–
Mutation factor		32.04	14.71																		
2-AA^g^	10	–	–	2634 ± 157.8	2446 ± 165.8	–	–	2830 ± 400.0	2500 ± 94.7	–	–	333 ± 5.7	275 ± 3.5	–	–	506 ± 50.9	381 ± 27.3	–	–	113 ± 12.2	119 ± 11.0
Mutation factor				97.56	94.08			23.78	20.83			25.62	25.00			38.92	31.75			2.17	2.90
Sodium azide^g^	1.5	–	–	–	–	505 ± 5.5	524 ± 9.5	–		567 ± 11.8	585 ± 32.8	–	–	–	–	–	–	–	–	–	–
Mutation factor						4.90	5.09			31.50	45.00										
ICR 191 acridine^g^	1	–	–	–	–	–	–	–	–	–	–	–	–	393 ± 20.7	5530 ± 95.6	–	–	–	–	–	–
Mutation factor														32.75	691.25						
MMS^g^	2.5	–	–	–	–	–	–	–	–	–	–	–	–	–	–	–	–	812 ± 7.8	373 ± 25.2	–	–
Mutation factor																		20.30	10.97		

Abbreviations: 2-AA, 2-aminoanthracene; LegH, leghemoglobin protein; MMS, methyl methanesulfonate; SD, standard deviation.

^a^n = 3 replicate plates.

^b^Dash lines (-): data not applicable.

^c^Rep 1—average from the main test, which followed the plate incorporation method.

^d^Rep 2—average from the confirmatory test, which followed the pre-incubation modification of the plate incorporation test.

^e^Mutation factor—calculated by dividing the mean revertant colony count by the mean revertant colony count for the corresponding concurrent vehicle control group.

^f^LegH levels correspond to LegH Prep concentrations of 23.384, 74, 233.84, 740, 2,338.4, 7,400, 23,384, and 74,000 µg/plate.

^g^Positive controls.

Leghemoglobin protein preparation did not cause a positive increase in the mean number of revertant colonies per plate with strains TA1535, TA1537, TA98, TA100, or WP2 uvrA in either the absence or the presence of S9 when using either the plate incorporation or the preincubation method (Table 1). Therefore, LegH Prep was nonmutagenic in the bacterial reverse mutation assay.

### Chromosome Aberration Assay in Human Peripheral BLD Lymphocytes

The objective of this in vitro assay was to evaluate the ability of LegH Prep to induce structural or numerical (polyploid or endoreduplicated) chromosome aberrations in HPBL. Human peripheral BLD lymphocytes cells were exposed to LegH Prep for 4 hours in the presence or absence of S9 (Experiment 1) or for 24 hours in the absence of S9 (Experiment 2). In each experiment, untreated and positive control values were within the historical control range indicating that the subject assay met the criteria for a valid test (Supplemental Table S4a-c).

In accordance with OECD guidelines, experiment 1 used the recommended concentrations of 100 to 5000 μg/mL LegH, which corresponded to 148 to 74 000 μg/mL LegH Prep (Supplemental Table S5). Mitotic index was evaluated first, since decreased mitotic index can inhibit the ability to evaluate chromosome aberrations. Although the mitotic index decreased to below 70% of the negative control at high concentrations of LegH Prep without S9 metabolic activation, no such decrease in the mitotic index was observed in the presence of S9 metabolic activation (Table 2). No significant difference in the proliferation index was observed under either condition (Table 3). Evaluation of chromosomal aberration tests using the mitotic index can be unreliable.^56^ However, in all cases, the mitotic index remained above the 45% of control threshold that is recommended for evaluation of structural and numerical chromosomal aberrations, and no test substance precipitation was observed. In experiment 1, no significant increase in cells with structural or numerical chromosome aberrations was observed up to 5000 μg/mL LegH, which was the maximum dose tested, both with and without S9 (Table 2).

**Table 2.** Human Peripheral Blood Lymphocytes Treated with LegH Prep—Chromosome Aberration Assay.

Treatment (w/v)	S9 activation	Treatment time	Mean mitotic index	Cells scored	Cells with aberrations	
					Including gaps	Excluding gaps
Experiment 1 (−S9)						
Control	−S9	4	40	300	17	10
LegH 500, μg/mL	−S9	4	35	300	18	12
LegH 1000, μg/mL	−S9	4	28	300	22	13
LegH 2500, μg/mL	−S9	4	23	300	18	12
LegH 5000, μg/mL	−S9	4	22	300	20	5
EMS 900, µg/mL	−S9	4	25	200	37	32
Experiment 1 (+S9)						
Control	+S9	4	38	300	23	11
LegH 1000, μg/mL	+S9	4	42	300	22	13
LegH 2500, μg/mL	+S9	4	32	300	15	6
LegH 5000, μg/mL	+S9	4	40	300	15	8
CPA 7.5, µg/mL	+S9	4	34	200	37	31
Experiment 2 (−S9)						
Control	−S9	24	60	300	13	7
LegH 100, μg/mL	−S9	24	52	300	18	8
LegH 200, μg/mL	−S9	24	57	300	20	10
LegH 500, μg/mL	−S9	24	42/P	300	10	6
LegH 1000, μg/mL	−S9	24	32/P	300	10	6
LegH 2000, μg/mL	−S9	24	16/P	ND	ND	ND
LegH 3000, μg/mL	−S9	24	8/P	ND	ND	ND
LegH 4000, μg/mL	−S9	24	23/P	ND	ND	ND
LegH 5000, μg/mL	−S9	24	25/P	ND	ND	ND
EMS 400, µg/mL	−S9	24	29	75	43	41

Abbreviations: EMS, Ethyl methanesulfonate; CPA, cyclophosphamide; ND, not determined; P, precipitate observed; LegH, leghemoglobin protein; LegH Prep, leghemoglobin protein preparation.

**Table 3.** Human Peripheral Blood Lymphocytes Treated With LegH Prep—Proliferation Index.

Treatment (LegH w/v)	S9 activation	Treatment time, hours	Proliferation index	Cells in mitosis number		
				1	2	3
Experiment 1 (−S9)						
Control	−S9	4	1.16	84	16	0
5000, μg/mL	−S9	4	1.09	91	9	0
Experiment 1 (+S9)						
Control	+S9	4	1.12	88	12	0
5000, μg/mL	+S9	4	1.07	93	7	0
Experiment 2 (−S9)						
Control	−S9	24	1.56	44	56	0
500, μg/mL	−S9	24	1.23	77	23	0
1000, μg/mL	−S9	24	1.12	88	12	0

Abbreviations: LegH, leghemoglobin protein; LegH Prep, leghemoglobin protein preparation.

The increased incubation time of experiment 2 resulted in precipitation of the test substance at concentrations of ≥500 µg/mL LegH. Additionally, the mitotic index values relative to the control decreased below the 45% threshold at concentrations >1000 μg/mL LegH. Therefore, only concentrations up to 1000 μg/mL LegH were evaluated for chromosome aberrations. The proliferation index values for 500 and 1000 µg/plate were 1.23 (79% relative to control) and 1.12 (72% relative to control), respectively (Table 3). This decrease was not a consequence of chromosome aberrations. In experiment 2, no significant increase in cells with structural or numerical chromosome aberrations was observed up to 1000 μg/mL LegH, which was the maximum dose evaluated, without metabolic activation (Table 2).

These results indicate that LegH Prep does not induce structural or numerical chromosome aberrations in either the nonactivated or the S9-activated test system. Therefore, LegH Prep is considered nonclastogenic in the in vitro mammalian chromosome aberration test using HPBL.

### Animal Feed Analytical Chemistry

In each of the in vivo studies described below, diets were prepared weekly using a food mixer and provided to the animals ad libitum. Each diet preparation was split into half, and 1 aliquot was stored at −20°C. The feed within the animal cages was replaced twice a week, which was a conservative approach implemented to minimize the amount of time that each formulation was held at room temperature. Dietary preparations were analyzed using HPLC to evaluate test substance (freeze-dried LegH Prep) homogeneity and stability and for concentration verification. The neat test substance served as the reference standard for each feed measurement. In each case, the analyte fell within acceptable parameters: <10% relative standard deviation of LegH Prep concentration between samples of the feed collected from top, middle, and bottom of the mixer, indicating homogeneous distribution; <10% change in LegH Prep concentration during diet presentation, indicating stability; and within 10% of the target concentration of LegH Prep, indicating accurate dosing. Detailed analytical chemistry HPLC results from the *28-Day Dietary Feeding Study in Rats* are presented in Supplemental Tables S6 to S9.

### Fourteen-Day Dietary Palatability and Range-Finding Study in Rats

A 14-day toxicology and palatability feeding study in rats was performed to assess the feasibility of oral administration of freeze-dried LegH Prep in the diet and to establish the dose range for the subsequent 28-day study. The test substance was administered in doses of 0, 3156, 6312, and 12,612 ppm (groups 1-4, respectively). These concentrations were selected to administer target doses of 0, 263, 525, and 1050 mg/kg/d LegH Prep, corresponding to active ingredient LegH doses of 0, 125, 250, and 500 mg/kg/d, based on the assumption of a 300 g rat consuming 25 g of food per day (Supplemental Table S10). The feed formulation was held constant throughout the study. The calculated nominal dietary intake levels were 0, 134, 269, and 531 mg/kg/d for groups 1 to 4 male rats and 0, 148, 296, and 592 mg/kg/d for groups 1 to 4 female rats, respectively. The animals are considered to have received acceptable dose levels.

### Mortality, clinical signs, body weight/food consumption

There were no mortalities during the course of the 14-day study. There were no clinical observations attributed to the administration of LegH Prep. In-life clinical signs included reported discoloration of the urine in the 6/6 group 4 males and 5/6 group 4 females on day 5. Due to its single-day occurrence and lack of occurrence in any other in-life study, this observation is likely due to rehydration of the test substance by animal urine, which would result in the formation of a red/brown COL. Without a correlation to clinical hematology or any other parameter, these findings are interpreted to be of no toxicological significance. There were no changes in mean food consumption, mean food efficiency, mean body weight, and mean daily body weight gain attributable to the administration of LegH Prep.

#### Pathology

There were no changes in hematology values attributable to the administration of LegH Prep. There were no LegH Prep-related macroscopic or microscopic findings. Mean absolute and relative organ-to-body weights for groups 2 to 4 were comparable to control group 1 throughout the study. These results suggest that LegH Prep would be well tolerated in a study of longer duration.

### Twenty-Eight-Day Dietary Feeding Study in Rats

A 28-day dietary feeding study in rats was performed to evaluate the potential subchronic toxicity of LegH Prep following continuous exposure of the test substance in the diet. A no observed adverse effect level (NOAEL) was sought for each sex. Due to the successful palatability of 500 mg/kg/d LegH in the previous 14-day feeding study, the maximum dose was increased to 750 mg/kg/d LegH. Administered doses of 0, 512, 1024, and 1536 mg/kg/d of freeze-dried LegH Prep corresponded to 0, 250, 500, and 750 mg/kg/d of LegH, respectively (Supplemental Table S11). The slight difference in correlation between LegH Prep dose levels and LegH concentrations compared to the previous 14-day study are due to the utilization of a different lot of freeze-dried LegH Prep test substance. The mean overall daily intake of the test substance in groups 1 to 4 male rats was 0, 234, 466, and 702 mg/kg/d LegH, respectively. The mean overall daily intake in groups 1 to 4 female rats was 0, 243, 480, and 718 mg/kg/d LegH, respectively. The animals are considered to have received acceptable dose levels.

#### Mortality, clinical signs, and body weight/food consumption

No mortalities were observed during this study. There were no clinical observations attributable to the administration of LegH Prep. There were no body weight, body weight gain, food consumption, or food efficiency findings considered attributable to LegH Prep administration (Tables 4–6). A statistically significant decrease (*P* < 0.01) in mean daily body weight gain was observed in group 2 females on days 14 to 21 (Table 4). This decrease was transient and was interpreted to have no toxicological relevance. Statistically significant increases (*P* < 0.05-0.01) were observed for mean daily food consumption in group 3 males on days 7 to 14 and in group 4 males on days 7 to 10, which were transient and without significant impact on body weight and were interpreted to be nontoxicologically relevant (Table 5). Mean food efficiency for the treated female rats in groups 2 to 4 was generally comparable to the control group 1 values throughout the study, with the exception of statistically significant increases (*P* < 0.01) in group 2 on days 14 to 21 that were transient and without significant impact on body weight and were interpreted to be nontoxicologically relevant (Table 6). These small but significant changes were all considered to be nontoxicologically relevant and nontest substance dependent.

**Table 4.** Summary of Mean Body Weights and Mean Body Weight Gain—28-Day Dietary Study.^a^

Mean body weight ± SD, mg/kg/d									Mean body weight gain ± SD, mg/kg/d								
LegH dose levels	0, mg/kg/d		250, mg/kg/d		500, mg/kg/d		750, mg/kg/d		LegH dose levels	0, mg/kg/d		250, mg/kg/d		500, mg/kg/d		750, mg/kg/d	
Study day	M	F	M	F	M	F	M	F	Study days	M	F	M	F	M	F	M	F
0	236.4 ± 6.1	174.1 ± 12.3	236.4 ± 6.1	174.4 ± 12.6	236.7 ± 7.0	175.6 ± 11.8	236.3 ± 6.7	174.3 ± 11.9	0-7	7.33 ± 1.35	3.46 ± 1.03	7.60 ± 0.94	3.80 ± 0.96	7.74 ± 1.40	4.06 ± 0.72	8.07 ± 1.16	3.57 ± 0.61
7	287.7 ± 14.0	198.3 ± 14.8	289.6 ± 11.1	201.0 ± 16.5	290.9 ± 14.3	240.0 ± 13.3	292.8 ± 12.2	199.3 ± 10.5	7-14	6.37 ± 0.77	2.93 ± 2.09	6.77 ± 1.30	2.50 ± 0.75	7.24 ± 1.49	2.81 ± 0.66	6.67 ± 1.13	3.14 ± 0.81
14	332.3 ± 16.5	218.8 ± 21.9	337.0 ± 18.4	218.5 ± 19.6	341.6 ± 24.2	223.7 ± 14.6	339.5 ± 18.6	221.3 ± 14.3	14-21	5.84 ± 1.15	2.91 ± 1.15	5.66 ± 0.91	1.51^b^ ± 0.82	6.13 ± 1.12	2.16 ± 1.03	5.77 ± 0.86	2.39 ± 0.96
21	373.2 ± 22.7	239.2 ± 24.0	376.6 ± 21.4	229.1 ± 19.4	384.5 ± 31.1	238.8 ± 19.4	379.9 ± 22.7	238.0 ± 13.1	21-28	3.07 ± 2.06	1.51 ± 1.03	3.19 ± 0.75	2.13 ± 0.85	3.67 ± 1.10	2.06 ± 0.63	3.66 ± 0.47	1.53 ± 0.79
28	394.7 ± 28.8	249.8 ± 24.0	398.9 ± 26.4	244.0 ± 23.3	410.2 ± 37.2	253.2 ± 17.7	405.5 ± 24.4	248.7 ± 12.4	0-28	5.65 ± 0.84	2.70 ± 0.60	5.80 ± 0.83	2.49 ± 0.53	6.20 ± 1.14	2.77 ± 0.30	6.04 ± 0.70	2.66 ± 0.34

Abbreviations: F, female; LegH, leghemoglobin protein; M, male; SD, standard deviation.

^a^n = 10 animals/sex/group.

^b^*P* < 0.01.

**Table 5.** Summary of Mean Daily Food Consumption—28-Day Dietary Study.^a^

Mean daily food consumption ± SD, mg/kg/d								
LegH dose levels	0, mg/kg/d		250, mg/kg/d		500, mg/kg/d		750, mg/kg/d	
Study day	M	F	M	F	M	F	M	F
0-3	18.73 ± 3.35	13.43 ± 2.05	18.73 ± 1.98	12.93 ± 2.21	18.80 ± 2.68	13.73 ± 1.74	19.03 ± 2.99	13.70 ± 2.56
3-7	28.03 ± 1.08	21.18 ± 1.24	28.60 ± 0.52	21.23 ± 1.13	28.23 ± 2.08	21.05 ± 1.31	28.63 ± 1.07	20.18 ± 0.77
0-7	24.04 ± 1.67	17.86 ± 0.98	24.37 ± 0.65	17.67 ± 1.06	24.19 ± 0.94	17.91 ± 1.02	24.51 ± 1.56	17.40 ± 0.82
7-10	26.30 ± 1.31	19.33 ± 2.23	27.10 ± 0.81	18.43 ± 0.54	27.80^b^ ± 1.97	19.30 ± 2.26	27.90^c^ ± 0.78	18.90 ± 0.96
10-14	26.55 ± 1.17	19.55 ± 1.59	27.25 ± 0.91	20.45 ± 2.02	27.88 ± 2.25	19.45 ± 1.12	27.45 ± 1.03	19.08 ± 1.11
7-14	26.44 ± 1.16	19.46 ± 1.83	27.19 ± 0.84	19.59 ± 1.29	27.84^c^ ± 2.12	19.39 ± 1.52	27.64 ± 0.84	19.00 ± 1.02
14-17	25.90 ± 0.89	19.27 ± 1.34	25.47 ± 1.83	19.40 ± 0.76	26.33 ± 2.71	18.47 ± 1.22	26.17 ± 0.82	18.73 ± 1.17
17-21	26.38 ± 0.97	19.88 ± 1.72	26.50 ± 0.77	20.08 ± 1.18	27.10 ± 2.31	19.35 ± 1.52	26.93 ± 0.86	19.13 ± 0.64
14-21	26.17 ± 0.93	19.61 ± 1.53	26.06 ± 1.19	19.79 ± 0.64	26.77 ± 2.42	18.97 ± 1.35	26.60 ± 0.67	18.96 ± 0.61
21-24	21.80 ± 0.77	15.90 ± 0.74	22.07 ± 1.03	16.23 ± 0.68	22.27 ± 1.61	15.97 ± 0.34	22.47 ± 0.66	15.63 ± 0.55
24-28	27.70 ± 1.04	20.70 ± 1.38	28.75 ± 1.21	21.33 ± 1.44	29.13 ± 1.92	21.08 ± 0.91	29.18 ± 1.28	20.45 ± 0.55
21-28	25.17 ± 0.89	18.64 ± 1.09	25.89 ± 1.10	19.14 ± 0.96	26.19 ± 1.73	18.89 ± 0.54	26.30 ± 0.83	18.39 ± 0.32
0-28	25.46 ± 0.91	18.89 ± 1.23	25.88 ± 0.87	19.05 ± 0.81	36.25 ± 1.58	18.79 ± 1.09	26.26 ± 0.90	18.44 ± 0.61

Abbreviations: F, female; LegH, leghemoglobin protein; M, male; SD, standard deviation.

^a^n = 10 animals/sex/group.

^b^*P* < 0.01.

^c^*P* < 0.05.

**Table 6.** Summary of Mean Food Efficiency and Mean Daily Dietary Intake of LegH Prep—28-Day Dietary Study.^a^

Mean food efficiency ± SD, mg/kg/d									Mean daily dietary intake of LegH Prep ± SD, mg/kg/d								
LegH dfose levels	0, mg/kg/d		250, mg/kg/d		500, mg/kg/d		750, mg/kg/d		LegH Prep dose levels	0, mg/kg/d		512, mg/kg/d		1024, mg/kg/d		1536, mg/kg/d	
Study day	M	F	M	F	M	F	M	F	Study days	M	F	M	F	M	F	M	F
0-7	0.304 ± 0.046	0.193 ± 0.055	0.312 ± 0.038	0.215 ± 0.052	0.319 ± 0.051	0.226 ± 0.037	0.329 ± 0.046	0.206 ± 0.038	0-7	0.0 ± 0.0	0.0 ± 0.0	485.4 ± 20.9	498.0 ± 43.5	966.5 ± 42.5	995.9 ± 29.2	1459.8 ± 103.0	1481.1 ± 115.0
7-14	0.241 ± 0.025	0.148 ± 0.093	0.248 ± 0.043	0.128 ± 0.037	0.258 ± 0.041	0.146 ± 0.033	0.241 ± 0.040	0.165 ± 0.040	7-14	0.0 ± 0.0	0.0 ± 0.0	540.5 ± 24.5	541.9 ± 49.9	1095.9 ± 53.5	1064.6 ± 39.2	1631.5 ± 78.9	1604.6 ± 116.7
14-21	0.223 ± 0.044	0.149 ± 0.057	0.217 ± 0.022	0.077^b^ ± 0.042	0.227 ± 0.026	0.112 ± 0.049	0.217 ± 0.031	0.126 ± 0.052	14-21	0.0 ± 0.0	0.0 ± 0.0	503.2 ± 30.7	518.8 ± 53.9	1007.2 ± 61.7	1015.1 ± 34.3	1513.7 ± 81.1	1537.2 ± 92.3
21-28	0.121 ± 0.040	0.080 ± 0.052	0.123 ± 0.032	0.111 ± 0.041	0.139 ± 0.040	0.109 ± 0.033	0.139 ± 0.019	0.083 ± 0.044	21-28	0.0 ± 0.0	0.0 ± 0.0	495.9 ± 33.2	482.4 ± 41.9	973.0 ± 49.1	994.0 ± 56.0	1473.9 ± 92.5	1460.2 ± 79.0
0-28	0.222 ± 0.031	0.142 ± 0.027	0.224 ± 0.028	0.131 ± 0.028	0.235 ± 0.034	0.147 ± 0.011	0.230 ± 0.027	0.144 ± 0.019	0-28	0.0 ± 0.0	0.0 ± 0.0	478.9 ± 24.7	497.8 ± 42.8	954.7 ± 36.0	983.4 ± 29.0	1438.2 ± 78.6	1470.4 ± 88.2

Abbreviations: F, female; LegH, leghemoglobin protein; LegH Prep, leghemoglobin protein preparation; M, male; SD, standard deviation.

^a^n = 10 animals/sex/group.

^b^*P* < 0.01.

#### Pathology

There were to no test substance-related changes in hematology parameters for male rats (Table 7). Statistically significant increase in red BLD cell, hematocrit, and hemoglobin values and absolute basophil counts for group 2 females, and decreased ARETs in group 3 females were nondose dependent and were interpreted to be within the expected biological variation and, therefore, not toxicologically relevant and not test substance dependent (Table 7). There were no test substance-related changes in coagulation parameters for female rats. A nondose-dependent increase in APTT was observed in groups 3 and 4 males. Due to its very slight magnitude and lack of correlating pathological or clinical finding, this change is considered nonadverse. There were no test substance-related changes in serum chemistry parameters for male rats (Table 8). Alkaline phosphatase was minimally decreased in a nondose-dependent manner for group 2 and group 4 females (Table 8). This minimal decrease was not correlated with concurrent clinical pathology or histopathology changes, and due to its limited clinical relevance, it is interpreted to have no toxicological significance and was not test substance dependent. Other differences in serum chemistry parameters that were statistically significant consisted of increased ALB and K values in group 3 males, decreased GLUC and chloride in groups 2 and 3 females, increased GLOB values in group 3 females, and increased CALC in groups 2 and 3 females. These were generally of small magnitude, lacked a response in a dose-dependent manner, and are interpreted to be within expected biological variation and considered to be of no toxicological relevance and non-test substance dependent. There were no test substance-related changes in urinalysis parameters for male or female rats (Table 9). Urine sediment analysis was performed for all animals and all results were within normal limits.

**Table 7.** Hematology and Coagulation—28-Day Dietary Study.^a^

Clinical pathology—hematology, mean ± SD									Historical means and ranges	
LegH dose levels	0, mg/kg/d		250, mg/kg/d		500, mg/kg/d		750, mg/kg/d			
Parameter	M	F	M	F	M	F	M	F	M	F
RBC, × 106/mL	7.72 ± 0.23	7.59 ± 0.24	7.60 ± 0.34	8.01^b^ ± 0.38	7.61 ± 0.35	7.86 ± 0.24	7.70 ± 0.27	7.63 ± 0.30	8.75, 5.07-10.04	8.26, 6.99-9.34
HGB, g/dL	15.6 ± 0.3	15.3 ± 0.5	15.4 ± 0.6	16.2^b^ ± 0.5	15.5 ± 0.6	15.7 ± 0.4	15.9 ± 0.4	15.5 ± 0.6	15.7, 10.5-17.4	15.4, 13.4-17.3
HCT, %	45.5 ± 0.9	43.6 ± 1.2	45.1 ± 1.5	45.9^b^ ± 1.2	45.1 ± 1.7	44.7 ± 1.3	45.9 ± 0.8	44.0 ± 1.7	46.1, 34.8-50.6	44.8, 38.0-49.4
MCV, fL	58.9 ± 1.0	57.5 ± 1.1	59.3 ± 2.3	57.4 ± 2.2	59.3 ± 1.5	56.8 ± 1.2	59.7 ± 1.9	57.7 ± 2.2	52.8, 47.5-68.6	54.3, 49.9-58.6
MCH, pg	20.3 ± 0.5	20.2 ± 0.3	20.3 ± 0.9	20.2 ± 0.7	20.4 ± 0.5	20.0 ± 0.5	20.6 ± 0.7	20.3 ± 0.7	17.9, 15.3-20.7	18.7, 17.0-20.8
MCHC, g/dL	34.4 ± 0.4	35.2 ± 0.7	34.2 ± 0.4	35.3 ± 0.3	34.4 ± 0.3	35.2 ± 0.4	34.5 ± 0.5	35.2 ± 0.5	34.0, 30.1-36.7	34.5, 32.5-36.5
RDW, %	12.1 ± 0.3	11.3 ± 0.4	12.5 ± 0.5	11.3 ± 0.5	12.5 ± 0.3	11.2 ± 0.3	12.3 ± 0.5	11.5 ± 0.5	13.3, 11.3-31.2	11.6, 10.1-13.1
PLT (×103/mL	1160 ± 121	1190 ± 108	1202 ± 69	1176 ± 127	1171 ± 76	1230 ± 115	1227 ± 185	1229 ± 114	990, 404-1799	1013, 448-1594
WBC (×103/mL)	13.00 ± 1.33	10.08 ± 1.70	14.41 ± 2.67	11.87 ± 1.75	11.13 ± 1.82	11.59 ± 3.35	13.45 ± 4.41	10.19 ± 3.72	11.94, 2.75-22.23	7.66, 2.41-17.04
ANEU, ×103/mL	1.91 ± 0.67	1.48 ± 0.30	1.99 ± 0.43	1.56 ± 0.58	1.75 ± 0.43	1.68 ± 0.85	1.57 ± 0.62	1.54 ± 1.10	2.11, 0.53-9.39	1.08, 0.31-4.08
ALYM, ×103/mL	10.49 ± 1.17	8.15 ± 1.58	11.79 ± 2.48	9.74 ± 1.43	8.86 ± 1.70	9.29 ± 2.71	11.29 ± 4.15	8.21 ± 2.88	9.12, 1.47-20.32	6.16, 1.79-12.84
AMON (×103/mL)	0.31 ± 0.10	0.25 ± 0.15	0.34 ± 0.11	0.29 ± 0.06	0.28 ± 0.05	0.33 ± 0.15	0.30 ± 0.10	0.22 ± 0.14	0.35, 0.09-0.93	0.20, 0.03-0.56
AEOS (×103/mL)	0.12 ± 0.04	0.11 ± 0.03	0.13 ± 0.08	0.13 ± 0.04	0.11 ± 0.04	0.15 ± 0.05	0.11 ± 0.05	0.12 ± 0.06	0.17, 0.00-0.88	0.13, 0.04-0.84
ABAS, ×103/mL	0.09 ± 0.03	0.04 ± 0.01	0.09 ± 0.04	0.07^b^ ± 0.03	0.07 ± 0.02	0.06 ± 0.03	0.10 ± 0.06	0.05 ± 0.04	0.06, 0.00-0.27	0.03, 0.00-0.15
ALUC ×103/mL	0.08 ± 0.03	0.05 ± 0.02	0.08 ± 0.03	0.07 ± 0.02	0.06 ± 0.02	0.07 ± 0.03	0.08 ± 0.04	0.05 ± 0.04	0.11, 0.00-0.47	0.06, 0.00-0.26
ARET (×103/mL)	232.6 ± 31.2	205.8 ± 33.9	235.8 ± 40.7	182.4 ± 32.9	246.3 ± 24.1	169.1^b^ ± 30.9	243.8 ± 41.1	184.2 ± 33.7	219.5, 98.6-1913.0	164.6, 27.7-277.2
PT, seconds	10.7 ± 0.3	10.0 ± 0.2	10.7 ± 0.4	9.8 ± 0.2	10.6 ± 0.2	10.0 ± 0.3	10.6 ± 0.2	9.8 ± 0.2	10.53, 9.5-12.1	9.9, 9.2-10.7
APTT, seconds	20.2 ± 2.4	21.9 ± 2.5	23.8 ± 5.3	20.0 ± 3.1	24.9^b^ ± 6.9	20.8 ± 5.0	24.9^b^ ± 6.9	19.4 ± 1.9	19.86, 13.5-54.8	19.0, 13.2-48.5

Abbreviations: ABAS, absolute basophils; AEOS, absolute eosinophils; ALUC, absolute leucocytes; ANEU, absolute neutrophils; ALYM, absolute lymphocytes; AMON, absolute monocytes; APTT, activated partial thromboplastin time; ARET, absolute reticulocyte count; F, female; HCT, hematocrit; HGB, hemoglobin concentration; LegH, leghemoglobin protein; M, male; MCH, mean corpuscular hemoglobin; MCHC, mean corpuscular hemoglobin concentration; MCV, mean corpuscular volume; PLT, platelet count; PT, prothrombin time; RBC, erythrocyte count; RDW, red cell distribution width; SD, standard deviation; WBC, white blood cell.

^a^n = 10 animals/sex/group.

^b^*P* < .05.

**Table 8.** Serum Chemistry—28-Day Dietary Study.^a^

Clinical pathology—serum chemistry mean ± SD									Historical means and ranges	
LegH dose levels	0, mg/kg/d		250, mg/kg/d		500, mg/kg/d		750, mg/kg/d			
Parameter	M	F	M	F	M	F	M	F	M	F
AST, U/L	73 ± 8	69 ± 6	76 ± 9	69 ± 10	79 ± 7	64 ± 8	78 ± 8	65 ± 6	95, 52-514	77, 46-460
ALT, U/L	29 ± 4	25 ± 4	28 ± 4	26 ± 5	28 ± 3	25 ± 6	30 ± 4	27 ± 5	39, 18-290	33, 13-283
SDH, U/L	8.2 ± 1.4	8.7 ± 2.2	8.1 ± 1.7	8.1 ± 1.2	8.4 ± 2.4	8.0 ± 0.9	8.0 ± 1.4	9.9 ± 2.5	9.1, 0.0-126.0	8.0, 0.2-42.7
ALKP, U/L	183 ± 24	137 ± 16	216 ± 29	107^b^ ± 19	216 ± 44	121 ± 29	205 ± 42	108^b^ ± 25	93, 43-183	54, 17-179
BILI, mg/dL	0.17 ± 0.02	0.18 ± 0.02	0.17 ± 0.02	0.19 ± 0.02	0.18 ± 0.02	0.20 ± 0.02	0.18 ± 0.02	0.19 ± 0.03	0.16, 0.09-0.26	0.18, 0.10-0.28
BUN, mg/dL	10 ± 1	12 ± 2	11 ± 1	11 ± 1	10 ± 1	12 ± 2	11 ± 2	12 ± 1	13, 8-24	14, 7-24
CREA, mg/dL	0.22 ± 0.01	0.28 ± 0.02	0.23 ± 0.02	0.26 ± 0.02	0.23 ± 0.02	0.27 ± 0.03	0.21 ± 0.02	0.26 ± 0.03	0.29, 0.16-0.48	0.35, 0.21-0.53
CHOL, mg/dL	76 ± 16	85 ± 11	73 ± 27	95 ± 19	72 ± 14	98 ± 19	67 ± 12	94 ± 22	79, 34-145	89, 35-225
TRIG, mg/dL	66 ± 17	37 ± 6	67 ± 13	38 ± 9	67 ± 17	46 ± 15	68 ± 26	35 ± 8	87, 18-196	52, 15-265
GLUC, mg/dL	95 ± 12	118 ± 15	100 ± 9	103^b^ ± 10	102 ± 13	104^b^ ± 10	98 ± 8	110 ± 14	123, 68-256	120, 82-174
TP, g/dL	6.0 ± 0.2	6.4 ± 0.3	6.1 ± 0.2	67 ± 0.4	6.2 ± 0.2	6.8 ± 0.3	6.0 ± 0.2	6.7 ± 0.4	6.3, 5.3-7.4	7.0, 5.7-8.5
ALB, g/dL	3.1 ± 0.1	3.5 ± 0.2	3.2 ± 0.1	3.7 ± 0.2	33^b^ ± 0.1	3.7 ± 0.2	3.2 ± 0.1	3.6 ± 0.3	3.3, 2.8-4.0	3.8, 1-5.0
GLOB, g/dL	2.9 ± 0.1	2.9 ± 0.1	2.8 ± 0.2	3.1 ± 0.2	2.9 ± 0.1	3.1^b^ ± 0.2	2.8 ± 0.2	3.0 ± 0.1	3.0, 2.2-3.9	3.2, 2.1-4.8
CALC, mg/dL	10.4 ± 0.2	10.5 ± 0.3	10.4 ± 0.2	10.9^b^ ± 0.3	10.4 ± 0.2	11.0^b^ ± 0.3	10.5 ± 0.2	10.7 ± 0.4	10.2, 8.1-11.8	10.5, 9.0-12.1
IPHS, mg/dL	8.6 ± 0.4	7.1 ± 0.5	8.7 ± 0.4	7.8 ± 0.6	8.8 ± 0.9	7.6 ± 0.4	8.6 ± 0.4	7.1 ± 0.8	6.6, 4.5-9.0	5.3, 2.6-7.8
Na, mmol/L	140.5 ± 4.2	140.3 ± 1.1	142.1 ± 0.6	140.6 ±	141.1 ± 0.7	140.3 ± 0.7	141.7 ± 0.8	140.2 ± 1.1	142.6, 127.6-168.8	141.2, 126.3-163.6
K, mmol/L	5.03 ± 0.25	4.56 ± 0.33	5.19 ± 0.26	4.63 ± 0.38	5.55^b^ ± 0.61	4.72 ± 0.21	5.10 ± 0.25	4.74 ± 0.38	5.35, 3.97-8.46	4.54, 3.37-7.07
Cl, mmol/L	100.8 ± 2.4	102.6 ± 1.2	102.0 ± 1.0	101.3^b^ ± 1.4	101.6 ± 0.8	101.1^b^ ± 1.0	101.7 ± 1.2	102.1 ± 1.1	103.5, 91.9-121.5	103.3, 92.8-119.1

Abbreviations: ALB, albumin; ALKP, alkaline phosphatase; ALT, serum alanine aminotransferase; AST, serum aspartate amino transferase; BUN, urea nitrogen; BILI, total bilirubin; CALC, calcium; CHOL, total cholesterol; Cl, chloride; CREA, creatinine; F, female; GLOB, globulin; GLUC, glucose; IPHS inorganic phosphorus; IPHS K, potassium; LegH, leghemoglobin protein; M, male; Na, sodium; SDH, sorbitol dehydrogenase; SD, standard deviation; SG, specific gravity; TRIG, triglycerides; TP, total serum protein UMTP, protein; URO, urobilinogen; UVOL, urine volume.

^a^n = 10 animals/sex/group.

^b^*P* < 0.05.

**Table 9.** Urinalysis—28-Day Dietary Study.^a^

Clinical pathology—urinalysis, mean ± SD									Historical means and ranges	
LegH dose levels	0, mg/kg/d		250, mg/kg/d		500, mg/kg/d		750, mg/kg/d			
Parameter	M	F	M	F	M	F	M	F	M	F
UVOL, mL	11.7 ± 8.2	7.8 ± 6.4	11.5 ± 9.8	6.8 ± 5.1	12.3 ± 7.3	6.5 ± 3.0	14.3 ± 7.7	6.6 ± 4.1	7.3, 0.3-36.0	5.4, 0.1-39.0
pH	6.5 ± 0.3	6.4 ± 0.4	6.5 ± 0.4	6.2 ± 0.4	6.6 ± 0.4	6.6 ± 0.6	6.6 ± 0.4	6.5 ± 0.6	6.4, 5.0-8.5	6.5, 5.0-8.5
SG	1.027 ± 0.019	1.037 ± 0.027	1.027 ± 0.015	1.035 ± 0.023	1.026 ± 0.015	1.028 ± 0.011	1.024 ± 0.019	1.030 ± 0.013	1.052, 1.007-1.100	1.047, 1.009-1.100
URO, EU/dL	0.03 ± 0.03	0.2 ± 0.0	0.2 ± 0.0	0.2 ± 0.0	0.3 ± 0.3	0.2 ± 0.0	0.2 ± 0.0	0.3 ± 0.3	0.3, 0.2-1.0	0.3, 0.2-1.0
UMTP, mg/dL	104 ± 49	43 ± 34	241 ± 265	41 ± 25	124 ± 80	34 ± 12	111 ± 97	44 ± 30	213, 18-1330	92, 6-7400

Abbreviations: F, female; LegH, leghemoglobin protein; M, male; SD, standard deviation; SG, specific gravity; UMTP, protein; URO, urobilinogen; UVOL, urine volume.

^a^n = 10 animals/sex/group.

There were no test substance-dependent effects observed during necropsy, organ weights, macroscopic evaluation, and microscopic evaluation in male and female rats, with a single exception of a distinct estrous cycle stage distribution in the female rats (Table 10). The estrous cycle consists of 4 stages: proestrus, estrus, metestrus, and diestrous. Each stage has characteristic reproductive organ weights and histopathology. At study termination, groups 2 and 4 females had an increased incidence of animals in metestrus and a decreased incidence of animals in estrus compared to groups 1 and 3. Consistent with the estrous cycle stage distribution, group 2 and 4 females also had decreased presence of fluid-filled uteri and dilated uterine lumens and decreased uterine weights compared to groups 1 and 3 females (Tables 11 and 12). All other microscopic findings at the study day 29/ 30 time point were also unrelated to administration of LegH Prep and can be observed in the age and strain of rats used in this study.^57,58^ Although the differences in estrous cycle stage distribution between groups was likely due to sampling and assessing estrous cycle distribution on a single day, rather than using a longitudinal study, a more extensive and rigorous longitudinal study was performed focusing on the potential effect of LegH Prep on the estrous cycle.

**Table 10.** Summary of Estrous Cycle Stage Distribution—28-Day Dietary Study.

Number of rats in each estrous cycle stage				
LegH dose levels	0, mg/kg/d	250, mg/kg/d	500, mg/kg/d	750, mg/kg/d
Parameter				
D	0	1	3	0
E	4	1	2	0
P	2	0	2	2
M	4	8	3	8

Abbreviations: D, diestrus; E, estrus; LegH, leghemoglobin protein; P, proestrus; M, metestrus.

**Table 11.** Summary of Mean Terminal Body Weights and Organ Weights—28-Day Dietary Study.^a^

Terminal body and organ weights ± SD (g)								
LegH dose levels	0, mg/kg/d		250, mg/kg/d		500, mg/kg/d		750, mg/kg/d	
Parameter	M	F	M	F	M	F	M	F
Terminal BW	367.5 ± 25.3	229.2 ± 22.3	372.5 ± 23.8	225.6 ± 22.7	384.0 ± 33.4	236.3 ± 14.5	379.3 ± 21.4	233.8 ± 11.9
Adrenal	0.0654 ± 0.0068	0.0717 ± 0.0067	0.0655 ± 0.0112	0.0713 ± 0.0089	0.0593 ± 0.0116	0.0664 ± 0.0092	0.0672 ± 0.0098	0.0737 ± 0.0093
Brain	2.141 ± 0.095	2.007 ± 0.093	2.143 ± 0.110	1.976 ± 0.099	2.186 ± 0.140	2.046 ± 0.077	2.152 ± 0.105	2.021 ± 0.049
Epididymides	1.032 ± 0.123	–	1.088 ± 0.083	–	1.035 ± 0.131	–	1.008 ± 0.100	–
Heart	1.195 ± 0.104	0.840 ± 0.092	1.254 ± 0.121	0.830 ± 0.057	1.272 ± 0.113	0.850 ± 0.034	1.219 ± 0.088	0.848 ± 0.065
Kidneys	2.641 ± 0.297	1.752 ± 0.164	2.678 ± 0.219	1.820 ± 0.177	2.789 ± 0.246	1.769 ± 0.140	2.800 ± 0.241	1.815 ± 0.101
Liver	11.218 ± 1.657	7.156 ± 0.720	11.182 ± 0.691	7.636 ± 1.037	12.317 ± 1.804	7.338 ± 0.512	12.093 ± 1.452	7.763 ± 0.548
Ovaries–oviduct	–	0.1309 ± 0.0173	–	0.172 ± 0.0172	–	0.1231 ± 0.0143	–	0.1364 ± 0.0150
Spleen	0.831 ± 0.125	0.498 ± 0.088	0.813 ± 0.107	0.518 ± 0.119	0.769 ± 0.053	0.507 ± 0.068	0.809 ± 0.105	0.513 ± 0.060
Testes	3.148 ± 0.531	–	3.381 ± 0.292	–	3.266 ± 0.251	–	3.272 ± 0.246	–
Thymus	0.5205 ± 0.1595	0.4343 ± 0.0998	0.5661 ± 0.1162	0.4654 ± 0.0741	0.5466 ± 0.1185	0.4762 ± 0.0967	0.5276 ± 0.1097	0.5218 ± 0.1127
Uterus	–	0.727 ± 0.247	–	0.457^b^ ± 0.061	–	0.615 ± 0.276	–	0.490^c^ ± 0.057

Abbreviations: BW, Body weight; F, female; LegH, leghemoglobin protein; M, male; SD, standard deviation.

^a^n = 10 animals/sex/group.

^b^*P* < 0.01.

^c^*P* < 0.05.

**Table 12.** Summary of Mean Relative Organ-to-Body Weights—28-Day Dietary Study.^a^

Organ-to-body weight ratios ± SD (g)								
LegH dose levels	0, mg/kg/d		250, mg/kg/d		500, mg/kg/d		750, mg/kg/d	
Parameter	M	F	M	F	M	F	M	F
Adrenal/TBW	0.1781 ± 0.0165	0.3139 ± 0.0265	0.1766 ± 0.0328	0.3168 ± 0.0336	0.1540 ± 0.0253	0.2812 ± 0.0372	0.1773 ± 0.0264	0.3157 ± 0.0399
Brain/TBW	5.846 ± 0.411	8.801 ± 0.545	5.766 ± 0.355	8.828 ± 0.852	5.722 ± 0.497	8.692 ± 0.686	5.682 ± 0.294	8.664 ± 0.492
Epididymides/TBW	2.8075 ± 0.2682	–	2.9351 ± 0.3125	–	2.7030 ± 0.3143	–	2.6712 ± 0.3544	–
Heart/TBW	3.251 ± 0.151	3.665 ± 0.189	3.362 ± 0.151	3.692 ± 0.171	3.315 ± 0.128	3.605 ± 0.178	3.214 ± 0.149	3.625 ± 0.163
Kidneys/TBW	7.184 ± 0.610	7.657 ± 0.412	7.199 ± 0.541	8.094 ± 0.639	7.274 ± 0.421	7.505 ± 0.657	7.387 ± 0.560	7.783 ± 0.602
Liver/TBW	30.549 ± 4.348	31.278 ± 2.212	30.052 ± 1.405	33.819 ± 2.693	31.962 ± 2.654	31.158 ± 2.883	31.839 ± 3.559	33.269 ± 2.772
Ovaries–oviduct/TBW	–	0.5727 ± 0.0669	–	0.5635 ± 0.0474	–	0.5222 ± 0.0643	–	0.5835 ± 0.0581
Spleen/TBW	2.256 ± 0.255	2.171 ± 0.300	2.199 ± 0.391	2.284 ± 0.384	2.012 ± 0.184	2.149 ± 0.291	2.139 ± 0.312	2.191 ± 0.206
Testes/TBW	8.549 ± 1.201	–	9.108 ± 0.971	–	8.564 ± 0.970	–	8.657 ± 0.885	–
Thymus/TBW	1.4134 ± 0.4037	1.8863 ± 0.3463	1.5209 ± 0.3105	2.0742 ± 0.3287	1.4171 ± 0.2319	2.0184 ± 0.4057	1.3939 ± 0.2919	2.2362 ± 0.4918
Uterus/TBW	–	3.159 ± 0.949	–	2.060^b^ ± 0.452	–	2.579 ± 1.063	–	2.103^c^ ± 0.277

Abbreviations: F, female; LegH, leghemoglobin protein; M, male; SD, standard deviation; TBW, terminal body weight.

^a^n = 10 animals/sex/group.

^b^*P* < .01.

^c^*P* < .05.

### Twenty Eight-Day Investigative Study in Rats With a 14-Day Predosing Estrous Cycle Determination

A 28-day dietary feeding study was performed with female rats to thoroughly evaluate the estrous cycle stage distributions, decreased presence of fluid-filled uteri and dilated uterine lumens, and decrease in uterine weights observed the group 2 and group 4 females in the previous 28-day dietary feeding study. To ensure all animals had normal estrous cyclicity prior to the 28-day dosing phase, estrous cycle stage was determined daily for all animals for 14 days. Additionally, estrous cycle stage was determined for all animals for the last 14 days of the 28-day dosing period. At study termination, reproductive organs were analyzed. Administered doses of 0 (group 1, control), 512 (group 2, low), 1024 (group 3, medium), and 1536 (group 4, high) mg/kg/d of freeze-dried LegH Prep correspond to 0, 250, 500, and 750 mg/kg/d of LegH, respectively (Supplemental Table S12). The same lot of freeze-dried LegH Prep was used for both 28-day feeding studies. The mean overall daily intake of the test substance in groups 1 to 4 female rats was 0, 250, 496, and 738 mg/kg/d LegH, respectively. The animals are considered to have received acceptable dose levels.

#### Mortality, clinical signs, and body weight/food consumption

No mortalities were observed during this study. There were no clinical observations attributable to the administration of LegH Prep. There were no body weight, body weight gain, food consumption, or food efficiency findings considered attributable to LegH Prep administration with the exception of a single incidental increase (*P* < 0.05) in mean daily body weight, mean food consumption, and mean food efficiency for group 2 animals on days 21 to 28. This increase was transient, nondose dependent, and interpreted to have no toxicological relevance.

#### Estrous cycle evaluation and pathology

There were no test substance-related changes in average estrus cycle length attributable to LegH Prep administration (Table 13). There were no macroscopic or microscopic findings related to the administration of LegH Prep. A single group 2 animal had prolonged estrus based on morphology of the ovaries (large atretic follicles, multiple corpus lutea at a similar state of atresia) and the presence of squamous metaplasia of the uterus. These findings were considered spontaneous and incidental due to the lack of similar findings at higher dose levels. One group 1 animal had large atretic follicles observed in both ovaries, and one group 4 animal had lutenized follicles (follicles with evidence of lutenization in the wall but which have not ovulated) in both ovaries. Both of these observations are reported as background findings in rats of the strain and age used in this study^59^ and were considered incidental because of their singular occurrences. There were no test substance-related changes in absolute or relative reproductive organ weight values in female rats treated with LegH Prep (Table 14). Longitudinal daily monitoring of estrous cycle stage demonstrated that, despite intrinsically normal estrous cycles, the distribution of estrous cycle stages on any given day can be markedly different from the within-rat distribution over time (Supplemental Figure S1).

**Table 13.** Estrous Cycles—28-Day Dietary Study With Predosing Estrous Cycle Determination.^a^

Mean number of estrous cycles ± SD				
LegH dose levels	0, mg/kg/d	250, mg/kg/d	500, mg/kg/d	750, mg/kg/d
Pre-test 0-13	2.3 ± 0.5	2.4 ± 0.6	2.3 ± 0.6	2.1 ± 0.5
Study days 29-42	2.3 ± 0.5	1.9 ± 0.5	2.1 ± 0.3	2.1 ± 0.4

Abbreviations: LegH, leghemoglobin protein; SD, standard deviation.

^a^n = 15 animals/group.

**Table 14.** Summary of Mean Terminal Body and Organ Weights and Organ Relative Weights—28-Day Dietary Study With Predosing Estrous Cycle Determination.^a^

Mean terminal body and organ weights and organ relative weights ± SD, g								
LegH dose levels	0, mg/kg/d		250, mg/kg/d		500, mg/kg/d		750, mg/kg/d	
Parameter	Mean	Relative	Mean	Relative	Mean	Relative	Mean	Relative
Terminal BW	249.9 ± 21.8	–	253.1 ± 23.1	–	253.3 ± 22.9	–	259.0 ± 29.6	–
Ovaries–oviduct	0.1311 ± 0.0174	0.5270 ± 0.0733	0.1343 ± 0.0209	0.5325 ± 0.0772	0.1234 ± 0.0128	0.4886 ± 0.0467	0.1370 ± 0.0156	0.5332 ± 0.0667
Uterus	0.604 ± 0.221	2.412 ± 0.841	0.547 ± 0.102	2.166 ± 0.390	0.570 ± 0.162	2.276 ± 0.731	0.703 ± 0.223	2.745 ± 0.957

Abbreviations: BW, body weight; LegH, leghemoglobin protein; SD, standard deviation.

^a^n = 15 animals/group.

### Pathology Peer Review

Because there were no test substance-dependent effects observed in the estrous cycle study, a pathology peer review was performed on the initial 28-day dietary feeding study to evaluate distinct estrous cycle stage distribution, decreased presence of fluid-filled uteri and dilated uterine lumens, and decrease in uterine weights observed the group 2 and group 4 females to ensure that these findings were not indicative of a perturbation of the female estrous cycle. The review pathologist evaluated the estrous cycle stage distribution and organ weight and histopathology in all female reproductive organs and corresponding macroscopic and microscopic observations noted by the study pathologist. The decreases in uterine weights, fluid-filled uteri, and dilated uterine lumen did not correlate with any adverse histopathological findings and are therefore interpreted to be nonadverse. The presence of both new and old ovarian corpora lutea in females from all groups indicated that all females were cycling normally. Following the peer review, the study pathologist and review pathologist reached a consensus that there were no test-substance-dependent effects on the female estrous cycle and reproductive organs.

## Discussion

Heme is ubiquitous in the human diet and has been consumed for thousands of years. Replacing the myoglobin that catalyzes the unique flavor chemistry of meat derived from animals with LegH from soy opens an opportunity to develop plant-based meats that deliver to consumers the pleasure they demand from animal-derived meats, with a small fraction of the environmental impact. Leghemoglobin protein preparation is manufactured using a *P pastoris* production strain that has been engineered to overexpress LegH. Following submerged fed-batch fermentation, the Pichia cells are lysed and the LegH is isolated using a filtration-based recovery process. The LegH Prep ingredient contains 6% to 9% LegH, which makes up at least 65% of the total protein fraction. The balance of the proteins is from the Pichia host. Leghemoglobin protein preparation is stabilized with NaCl and sodium ascorbate. A complete analysis of the LegH Prep specifications and chemical composition is provided in Supplemental Table S1.

A previous study evaluating the safety of LegH Prep in food used in silico approaches, such as literature searches for reports of allergenicity or toxicity and extensive sequence homology comparisons to databases of known allergens and toxins.^30^ That study also analyzed sensitivity to pepsin digestion in an in vitro simulated gastric fluid. Collectively, the previous work concluded that food products containing LegH Prep posed a low risk of allergenicity and toxicity to consumers.^30^ In this study, we evaluated the safety profile of LegH Prep through a series of in vitro and in vivo studies.

The Pichia-derived LegH Prep was nonmutagenic in the bacterial reverse mutation test, which evaluated 5 strains of bacteria and 8 different concentrations of LegH Prep up to a maximum dose of 5000 μg LegH/plate. Similarly, LegH Prep was nonclastogenic in the chromosomal aberration test, which evaluated chromosomal rearrangements in HPBL following 4-hour (with and without metabolic activation) and 24-hour (without metabolic activation) incubations with LegH Prep. These assays tested LegH concentrations up to 5000 μg/mL for the 4-hour incubations. Due to test substance precipitation and decreased percent mitotic index, 1000 μg/mL LegH was the maximum dose evaluated for the 24-hour incubation. Together, these results demonstrate that LegH Prep is nonmutagenic and nonclastogenic under the in vitro conditions tested.

To evaluate the in vivo safety profile for potential systemic toxicity, a 28-day feeding study was conducted in rats in which LegH Prep was administered in the diet. There were no LegH Prep-dependent effects observed with the exception of a distinct estrous cycle stage distribution in groups 2 and 4 females at study termination. Groups 2 and 4 females had an increased incidence of the metestrus stage of the estrous cycle (Table 10), decreased presence of fluid filled uteri and dilated uterine lumens, and decreased uterine weight compared to groups 1 and 3 females (Tables 11 and 12). However, the correlation between estrous cycle stage and reproductive organ weight and pathology for each animal was consistent with published literature on normal healthy rats.^60^ Therefore, although the estrous cycle stage distribution was different between the groups, there were no data to suggest an adverse impact on the health of the female animals; the presence of both new and old ovarian corpora lutea indicated normal estrous cyclicity.^59^ Without evidence of an adverse effect in the female ovary or uterus pathology, the decrease in relative and absolute uterine weights in groups 2 and 4 females was interpreted to be nonadverse. Moreover, decreased uterine weight is normal for animals in the metestrus stage of the estrous cycle.^61^

To thoroughly evaluate the estrous cycle stage distributions, decreased presence of fluid-filled uteri and dilated uterine lumens, and decrease in uterine weights observed the group 2 and group 4 females, an in-depth follow-up 28-day dietary feeding study in female rats was performed with longitudinal estrous cycle monitoring and evaluation of reproductive organ weights, gross necropsy, and histopathology. The results demonstrated that LegH Prep had no impact on the estrous cycle length, distribution, or female reproductive organ health when administered at dose levels up to 750 mg/kg/d LegH for 28 days (Tables 13 and 14). Despite intrinsically normal estrous cycles, different estrous cycle stage distributions were observed between groups on any given day (Supplemental Figure S1). This highlights the importance of longitudinal estrous cycle monitoring to evaluate estrous cyclicity. For example, if the estrous cycle stages were only monitored on a single day, a completely different conclusion would have been drawn regarding the test substance effect on estrous cycle if the animals had been analyzed, for example, on day 18 of the dosing period compared to day 21 (Supplemental Figure S1). The single-day sampling artifact readily accounts for the increased incidence of metestrus observed in the initial 28-day dietary feeding study. Moreover, a pathology peer review of the original 28-day study resulted in a consensus between the study pathologist and review pathologist that LegH Prep did not affect the estrous cycle. This is consistent with the lack of any scientific literature identifying phytoestrogens in Pichia as well as the absence of any published incidence of heme proteins affecting the estrous cycle.

The intake of LegH was assessed as the EDI and a 90th percentile user, representing the worst-case scenario in the consumption of LegH as a flavor catalyst in meat replacement products. Safety was assessed as a function of the actual exposure, and feeding study dose levels were chosen purposefully to reflect the actual exposure situations that would be encountered by a user. LegH Prep will not be sold to consumers as an individual ingredient and will instead be included in plant-based meat products at a level not exceeding 0.8% LegH. Together, these systemic toxicity and reproductive health feeding studies in rats established an NOAEL of 750 mg/kg/d LegH for both sexes, which was the maximum dose administered. The acceptable daily intake (ADI) is calculated by dividing the NOAEL by an acceptable uncertainty factor. In the absence of extenuating circumstances, the food additive regulations 21 C.F.R. 170.22 recommend a factor of 100. The ADI for soy leghemoglobin is 750/100 or 7.5 mg/kg/d. The 90th percentile EDI for soy leghemoglobin is 6.67 mg/kg/d. Based on FDA guidelines, since the EDI is lower than the ADI, these results suggest there are no safety concerns.^62^ Collectively, these in vitro and in vivo results suggest that LegH Prep, containing both soy LegH and Pichia proteins from the production host, raise no issues of toxicological concern under the conditions tested.

Creating safe, delicious plant-based meats to replace animal-derived meats in the diet is critical to reducing and eventually eliminating the environmental impact of the animal farming industry. Impossible Foods Inc has shown that plant-based meat containing up to 0.8% LegH delivers flavors and aromas that are characteristic of animal-derived meat.^11^ This study established an NOAEL of 750 mg/kg/d LegH, which is over 100 times higher than the 90th percentile EDI. This maximum dose is equivalent to an average-sized person (60 kg) consuming 5625 g (12 lbs) of plant-based ground beef analogue with 0.8% LegH per day. Thus, the results of the studies presented in this article raise no questions of toxicological concern under the conditions tested for LegH Prep, which is intended for use in ground beef analogue products at levels up to 0.8% LegH.

## Supplemental Material

Supplemental Material, DS1_IJT_10.1177_1091581818766318 - Safety Evaluation of Soy Leghemoglobin Protein Preparation Derived From *Pichia pastoris*, Intended for Use as a Flavor Catalyst in Plant-Based Meat Click here for additional data file. Supplemental Material, DS1_IJT_10.1177_1091581818766318 for Safety Evaluation of Soy Leghemoglobin Protein Preparation Derived From *Pichia pastoris*, Intended for Use as a Flavor Catalyst in Plant-Based Meat by Rachel Z. Fraser, Mithila Shitut, Puja Agrawal, Odete Mendes, and Sue Klapholz in International Journal of Toxicology

## Supplementary Material

Supplementary material

## Note

1. Datamonitor estimates the US meat analogue volume was 53M kg in 2009.
