# Supplementary material for: Safety Evaluation of Soy Leghemoglobin Protein Preparation Derived From Pichia pastoris, Intended for Use as a Flavor Catalyst in Plant-Based Meat
Source: Int J Toxicol. 2018 Apr 11;37(3):241–62. doi: 10.1177/1091581818766318 (PMC5956568; doi:10.1177/1091581818766318)

Supplementary Material

Table S1. LegH Prep specifications and batch analyses from two independent production runs. PP-PGM2-16-015-101 was used for all genotoxicology tests. PP-PGM2-16-088-101 was freeze dried and used for the *28-Day Dietary Feeding Study in Rats* and *28-Day Investigative Study with a 14-Day Pre-Dosing Estrous Cycle Determination.*

	**Specifications**	**PP-PGM2-16-015-101**	**PP-PGM2-16-088-101**
**Soy Leghemoglobin Protein (w/w)**	6 – 9%	6.74%	6.39%
**Soy Leghemolglobin Protein Purity (w/w)[2]**	≥65%	82%	71%
**Fat (w/w)**	≤2%	0.05%	<0.01%
**Carbohydrates (w/w)**	≤4%	1.72%	0.99%
**Ash (w/w)**	≤4%	1.87%	0.67%
**Solids (w/w)[3]**	≤24%	14.85%	12.55%
**Moisture (w/w)**	≥76%	85.15%	87.45%
**pH**	6.5 – 8.5	7.19	7.19
**Lead (ppm)**	<0.4	<0.01	<0.01
**Arsenic (ppm)**	<0.05	0.01	<0.01
**Mercury (ppm)**	<0.05	<0.005	<0.005
**Cadmium (ppm)**	<0.2	<0.001	<0.001
**Aerobic plate count (CFU/g) [4]**	<10^4	<10	<10
***E. coli 0157:H7* [5]**	Absent by test	Absent by test	Absent by test
***Salmonella spp.*[6]**	Absent by test	Absent by test	Absent by test
***Listeria monocytogenes* [7]**	Absent by test	Absent by test	Absent by test

[1] Soy leghemoglobin protein may exceed 9% if additional water (moisture) is removed during the concentration step of the manufacturing process. Additional concentration (i.e. less water) does not change the composition of the dry solids.

[2] The balance of the proteins in the preparation is residual Pichia proteins.

[3] Percent solids specification is based on the sum of the maximum concentrations of total protein, fat, carbohydrates and ash. Maximum total protein was calculated as the maximum soy leghemoglobin protein concentration divided by the minimum soy leghemoglobin protein purity.

[4] AOAC OMA 990.12

[5] AOAC RI 020801

[6] AOAC OMA 2011.03

[7] AOAC OMA 2010.02

n/a = not applicable

Table S2. Dose levels for *Bacterial Reverse Mutation Assay (Ames Test).*

**Dose Number**	**LegH Prep (µg/plate)**	**Soy Leghemoglobin Active Ingredient (µg/plate)**
0	0	0
1	23.384	1.58
2	74	5.0
3	233.84	15.8
4	740	50
5	2338.4	158
6	7400	500
7	23,384	1580
8	74,000	5,000[A]

[A]The OECD standard limit dose

Table S3. Bacterial reverse mutation assay historical control data maintained by PSL from 2015.

Plate Incorporation Method - Revertants Per Plate							
**Strain**	**Treatment**	**Dose (µg/plate)**	**S9**	**Mean**	**SD**	**Min**	**Max**
TA1535	Sodium Azide	1.5	-	618	91	359	1192
TA1537	ICR 191 Acridine	1	-	1136	1437	119	6388
TA98	Daunomycin	6	-	938	343	350	1500
TA100	Sodium Azide	1.5	-	600	126	394	1003
E. Coli	MMS	2.5	-	634	101	386	846
TA1535	2-AA	10	+	267	86	85	636
TA1537	2-AA	10	+	280	99	42	542
TA98	2-AA	10	+	2321	971	83	3915
TA100	2-AA	10	+	2377	806	976	4169
E. Coli	2-AA	10	+	125	30	63	196
TA1535	Sterile Water	N/A	-	13	2	7	21
TA1537	Sterile Water	N/A	-	12	4	6	25
TA98	Sterile Water	N/A	-	28	8	16	49
TA100	Sterile Water	N/A	-	130	16	104	155
E. Coli	Sterile Water	N/A	-	45	7	29	57
TA1535	Sterile Water	N/A	+	13	1	9	20
TA1537	Sterile Water	N/A	+	15	3	8	28
TA98	Sterile Water	N/A	+	29	5	18	40
TA100	Sterile Water	N/A	+	145	14	116	170
E. Coli	Sterile Water	N/A	+	59	13	31	81

Table S3 (continued). Bacterial reverse mutation assay historical control data maintained by PSL from 2015.

Pre-Incubation Method - Revertants Per Plate							
**Strain**	**Treatment**	**Dose (µg/plate)**	**S9**	**Mean**	**SD**	**Min**	**Max**
TA1535	Sodium Azide	1.5	-	622	71	478	831
TA1537	ICR 191 Acridine	1	-	3227	1227	875	5700
TA98	Daunomycin	6	-	602	345	146	1227
TA100	Sodium Azide	1.5	-	539	166	138	904
E. Coli	MMS	2.5	-	509	143	313	808
TA1535	2-AA	10	+	293	64	64	391
TA1537	2-AA	10	+	260	107	112	541
TA98	2-AA	10	+	2384	938	506	3530
TA100	2-AA	10	+	2388	583	1308	3620
E. Coli	2-AA	10	+	128	29	60	188
TA1535	Sterile Water	N/A	-	16	3	8	23
TA1537	Sterile Water	N/A	-	14	5	5	23
TA98	Sterile Water	N/A	-	29	7	14	46
TA100	Sterile Water	N/A	-	118	18	83	143
E. Coli	Sterile Water	N/A	-	46	10	30	67
TA1535	Sterile Water	N/A	+	12	2	8	19
TA1537	Sterile Water	N/A	+	14	5	6	26
TA98	Sterile Water	N/A	+	35	7	23	50
TA100	Sterile Water	N/A	+	125	16	88	147
E. Coli	Sterile Water	N/A	+	53	9	36	76

Table S4a. Historical laboratory control data of the negative control (short term treatment) from 2010 to 2015. The historical data without metabolic activation comprise the 4h and the 24 h treatment interval.

	NC Number of Aberrant Cells			
	- Metabolic Activation		+ Metabolic Activation	
	+ Gaps	- Gaps	+ Gaps	- Gaps
Mean [%]	3.4	1.5	3.1	1.3
SD [%]	1.57	0.84	1.63	0.82
RSD [%]	46.4	55.6	52.3	62.0
Min [%]	0.5	0.0	0.0	0.0
Max [%]	6.5	3.0	9.7	3.7
n	35	35	62	62

NC: Negative control
Mean: mean number of aberrant cells
SD: standard deviation
RSD: relative standard deviation
Min: minimum number of aberrant cells
Max: maximum
n: number of assays

Table S4b. Historical laboratory control data of the negative control (long term treatment) from 2010 to 2015.

	NC Number of Aberrant Cells	
	- Metabolic Activation	
	+ Gaps	- Gaps
Mean [%]	3.0	1.1
SD [%]	1.54	0.90
RSD [%]	51.4	79.1
Min [%]	0.5	0.0
Max [%]	6.9	4.2
n	32	32

NC: Negative control
Mean: mean number of aberrant cells
SD: standard deviation
RSD: relative standard deviation
Min: minimum number of aberrant cells
Max: maximum
n: number of assays

Table S4c. Historical laboratory control data of the positive control from 2010 to 2015. The historical data without metabolic activation comprise the 4h and the 24 h treatment interval.

	PC Number of Aberrant Cells			
	- Metabolic Activation		+ Metabolic Activation	
	+ Gaps	- Gaps	+ Gaps	- Gaps
Mean [%]	17.0	14.4	17.2	14.6
SD [%]	6.74	6.47	4.58	4.15
RSD [%]	39.7	45.0	26.6	28.4
Min [%]	9.5	7.0	31.0	26.7
Max [%]	41.0	38.1	31.0	26.7
n	68	68	62	62

PC: Positive control (EMS **without** metabolic activation, CPA **with** metabolic activation)
Mean: mean number of aberrant cells
SD: standard deviation
RSD: relative standard deviation
Min: minimum number of aberrant cells
Max: maximum
n: number of assays

Table S5. Dose levels for *Chromosome Aberration Assay in Human Peripheral Blood Lymphocytes (HPBL)***.**

**Test**	**Dose Group**	**LegH Prep (μg/mL)**	**Soy Leghemoglobin Active Ingredient (μg/mL)**
Experiment I 4 hr Treatment Without Metabolic Activation	C	0	0
	2	7400	500
	3	14,800	1000
	4	37,000	2500
	5	74,000	5000
Experiment I 4 hr Treatment With Metabolic Activation	C	0	0
	3	14,800	1000
	4	37,000	2500
	5	74,000	5000
Experiment II 24 hr Treatment Without Metabolic Activation	C	0	0
	1	1480	100
	2	2960	200
	3	7400	500
	4	14,800	1000

Table S6. Neat test substance stability at -20 °C during *28-Day Dietary Feeding Study in Rats*. Percent recovery was measured for samples of neat test article collected immediately prior to mixing in the diet for the initial, middle, and final feed preparations.

Sampling Day	Measured Recovery (%)	% Change[1]	Overall Stability (%)
Day 0 (Initial)	94.96%	0.00%	100.00%
Day 14 (Middle)	95.29%	0.35%	100.35%
Day 21 (Final)	90.88%	-4.30%	95.70%

[1] $\frac{\text{Final Sample} - \text{Initial Sample}}{\text{Initial Sample}} \text{ x } 100$

Table S7. LegH Prep stability in the diet at room temperature during *28-Day Dietary Feeding Study in Rats*. The initial feed formulation (week 1) was stored at room temperature and sampled at days 0, 4, 7, and 10. The concentration of LegH Prep was determined by HPLC. Throughout the in-life phase of the *28 Day Feeding Study in Rats*, fresh feed was formulated with test article once a week. Half of each formulation was presented to the animals immediately and the remaining half was stored at -20C. After four days, the diet was replaced with the thawed second installment to minimize total time of the formulated feed at room temperature. The test article was stable in the feed for up to 10 days at room temperature, which well exceeds the 4 day stability requirement of this study.

Day[1]	Group	Target Concentration (ppm)	Measured Concentration (ppm)	% of Target[2]
0	1 (BO)	0	ND	NA
	2 (M)	4373	4508	103.08%
	2 (F)	4711	4645	98.61%
	3 (M)	8746	7951	90.91%
	3 (F)	9422	9034	95.89%
	4(M)	13118	12265	93.50%
	4(F)	14133	12808	90.62%
4	1 (BO)	0	ND	NA
	2 (M)	4373	4207	96.20%
	2 (F)	4711	4471	94.90%
	3 (M)	8746	8238	94.19%
	3 (F)	9422	8918	94.65%
	4(M)	13118	12097	92.22%
	4(F)	14133	13191	93.33%
7	1 (BO)	0	ND	NA
	2 (M)	4373	4202	96.09%
	2 (F)	4711	4468	94.84%
	3 (M)	8746	8200	93.76%
	3 (F)	9422	8728	92.63%
	4(M)	13118	12423	94.70%
	4(F)	14133	13547	95.85%
10	1 (BO)	0	ND	NA
	2 (M)	4373	3968	90.74%
	2 (F)	4711	4693	99.63%
	3 (M)	8746	8453	96.65%
	3 (F)	9422	8836	93.78%
	4(M)	13118	12825	97.77%
	4(F)	14133	13762	97.38%

NA = Not Applicable; ND = Not Detected; BO = Both Male and Female; M = Male; F = Female

[1] Days relative to the initial diet preparation.

[2] % of Target = Measured Conc. (ppm) / Target Conc. (ppm) x 100

Table S8. LegH Prep homogeneity in the diet administered in *28-Day Dietary Feeding Study in Rats*. From the initial diet formulation (week 1), samples were collected from the top, middle, and bottom of the mixer. LegH Prep concentration was determined by HPLC. <10% RSD between the top, middle, and bottom samples for each formulation indicates that homogeneous mixing was achieved for all feed preparations.

Day[1]	Group	Sample Location	Target Concentration (ppm)	Measured Concentration (ppm)	% of Target[2]	Average % of Target	RSD (%)
0	1 (BO)	Middle	0	ND	NA	NA	NA
	2 (M)	Top	4373	4302	98.38%	95.87%	2.92%
		Middle		4061	92.86%		
		Bottom		4215	96.38%		
	2 (F)	Top	4711	4853	103.01%	98.01%	4.77%
		Middle		4583	97.28%		
		Bottom		4416	93.74%		
	3 (M)	Top	8746	8636	98.74%	95.40%	3.09%
		Middle		8145	93.13%		
		Bottom		8250	94.33%		
	3 (F)	Top	9422	9669	102.62%	97.71%	5.50%
		Middle		9284	98.53%		
		Bottom		8666	91.98%		
	4 (M)	Top	13118	12226	93.20%	97.85%	5.24%
		Middle		13558	103.35%		
		Bottom		12724	97.00%		
	4 (F)	Top	14133	14567	103.07%	98.64%	5.57%
		Middle		14183	100.35%		
		Bottom		13072	92.49%		

NA = Not Applicable; ND = Not Detected
BO = Both Male and Female; M = Male; F = Female
[1] Day relative to initial dietary preparation
[2] % of Target = Measured Conc. (ppm) / Target Conc. (ppm) x 100

Table S9. LegH Prep concentration verification for dietary preparations administered in *28-Day Dietary Feeding Study in Rats*. Feed samples from the middle of the mixer were collected from the initial (day 0), middle (day 7), and final (day 21) feed preparations. LegH Prep concentration was determined by HPLC. The target concentration of test article in the feed was adjusted weekly based on average animal weight and the previous week's food consumption in order to achieve the desired mg/kg dose of active ingredient. All measured concentrations were within 10% of the target dose, indicating that the feed contained the desired dose level.

Day[1]	Group	Target Concentration (ppm)	Measured Concentration (ppm)	% of Target[2]
0[3]	1 (BO)	0	ND	NA
	2 (M)	4373	4061	92.86%
	2 (F)	4711	4583	97.28%
	3 (M)	8746	8145	93.13%
	3 (F)	9422	9284	98.53%
	4(M)	13118	13558	103.35%
	4(F)	14133	14183	100.35%
7	1 (BO)	0	ND	NA
	2 (M)	6093	6158	101.06%
	2 (F)	5824	5326	91.45%
	3 (M)	12318	12189	98.96%
	3 (F)	11664	11408	97.81%
	4(M)	18362	19409	105.70%
	4(F)	17567	17238	98.13%
21	1 (BO)	0	ND	NA
	2 (M)	7407	6906	93.24%
	2 (F)	5925	5498	92.80%
	3 (M)	14727	14292	97.05%
	3 (F)	12901	12612	97.76%
	4(M)	21943	20786	94.73%
	4(F)	19281	18829	97.65%

NA = Not Applicable; ND = Not Detected
BO = Both Male and Female; M = Male; F = Female
[1] Days relative to the initial diet preparation.
[2] % of Target = Measured Conc. (ppm) / Target Conc. (ppm) x 100.
[3] As part of the homogeneity analysis.

Table S10. Dose levels for *14-Day Dietary Palatability and Range Finding Study in Rats.* The same feed formulation was used for both sexes on week 1 and week 2.

Group	No. Animals/ Group (Male/ Female)	Dietary Concentration of Freeze-Dried LegH Prep (ppm)	Dietary Concentration of Soy Leghemoglobin Active Ingredient (ppm)[A]	Target Exposure of Freeze-Dried LegH Prep (mg/kg/day)[B]	Target Exposure of Soy Leghemoglobin Active Ingredient (mg/kg/day)[A,B]	% of Target[C]
		Weeks 1-2				
1	6/6	0	0	0	0	NA
2	6/6	3156	1500	263	125	97.1%
3	6/6	6312	3000	525	250	97.7%
4	6/6	12,612	6000	1050	500	92.8%

[A] Based on 47.6% soy leghemoglobin active ingredient present within freeze-dried LegH Prep (PP-PGM2-16-081-301).
[B] Target mg/kg/day are estimated based on a 300 gram rat consuming 25 grams of diet per day
[C] % of Target = Measured Conc. (ppm) / Target Conc. (ppm) x 100 from the week 1 diet preparation.
NA = not applicable

Table S11. Dose levels for *28-Day Dietary Feeding Study in Rats.* Freeze-dried LegH Prep concentration in the feed was adjusted every week based on average animal weight and food consumption in order to achieve the target exposure (mg/kg/day) of soy leghemoglobin active ingredient.

Group	No. Animals/Group (Male/Female)	Dietary Concentration of Freeze-Dried LegH Prep (ppm)				Dietary Concentration of Soy Leghemoglobin Active Ingredient (ppm)[A]				Target Exposure of Freeze-Dried LegH Prep (mg/kg/day)[B]	Target Exposure of Soy Leghemoglobin Active Ingredient (mg/kg/day)[A,B]	% of Target[C]
		Week 1	Week 2	Week 3	Week 4	Week 1	Week 2	Week 3	Week 4	Weeks 1-4		
1	10 Male/Female	0	0	0	0	0	0	0	0	0	0	NA
2	10 Male	4373	6093	6346	7407	2134	2973	3097	3615	512	250	92.86%
	10 Female	4711	5824	5724	5925	2299	2842	2793	2891	512	250	97.28%
3	10 Male	8746	12,318	12,579	14,727	4268	6011	6139	7187	1024	500	93.13%
	10 Female	9422	11,664	11,830	12,901	4598	5692	5773	6296	1024	500	98.53%
4	10 Male	13,118	18,362	18,894	21,943	6402	8961	9220	10,708	1536	750	103.35%
	10 Female	14,133	17,567	17,866	19,281	6897	8573	8719	9409	1536	750	100.35%

[A] Based on 48.8% soy leghemoglobin active ingredient present within freeze-dried LegH Prep (PP-PGM2-16-088-301).

[B] Formulated each week based on average weight/group/sex and food consumption.

[C] % of Target = Measured Conc. (ppm) / Target Conc. (ppm) x 100 from the week 1 diet preparation.

NA = not applicable

Table S12. Dose levels for *28-Day Investigative Study with a 14-Day Pre-Dosing Estrous Cycle Determination.* Freeze-dried LegH Prep concentration in the feed was adjusted every week based on average animal weight and food consumption in order to achieve the target exposure (mg/kg/day) of soy leghemoglobin active ingredient.

Group	No. Animals/Group (Female)	Dietary Concentration of Freeze-Dried LegH Prep (ppm)				Dietary Concentration of Soy Leghemoglobin Active Ingredient (ppm)[B]				Target Exposure of Freeze-Dried LegH Prep (mg/kg/day)[A]	Target Exposure of Soy Leghemoglobin Active Ingredient (mg/kg/day)[A,B]	% of Target[C]
		Week 1	Week 2	Week 3	Week 4	Week 1	Week 2	Week 3	Week 4	Week 1-4		
1	15	0	0	0	0	0	0	0	0	0	0	NA
2	15	5863	5723	6082	6453	2861	2793	2968	3149	512	250	93.8%
3	15	11,354	11,686	12,288	12,683	5541	5703	5997	6189	1024	500	93.9%
4	15	16,936	17,815	19,021	19,788	8265	8694	9282	9657	1536	750	93.9%

[A] Based on 48.8% soy leghemoglobin active ingredient present within freeze-dried LegH Prep (PP-PGM2-16-088-301).
[B] Formulated each week based on average weight/group/sex and food consumption.
[C] % of Target = Measured Conc. (ppm) / Target Conc. (ppm) x 100 from the week 1 diet preparation.
NA = not applicable

Figure S1. Number of rats in the estrus phase of the estrous cycle for the first 10 rats within Groups 1 and 4 on each day. Data are from the 28-day dietary study with pre-dosing estrous cycle determination.

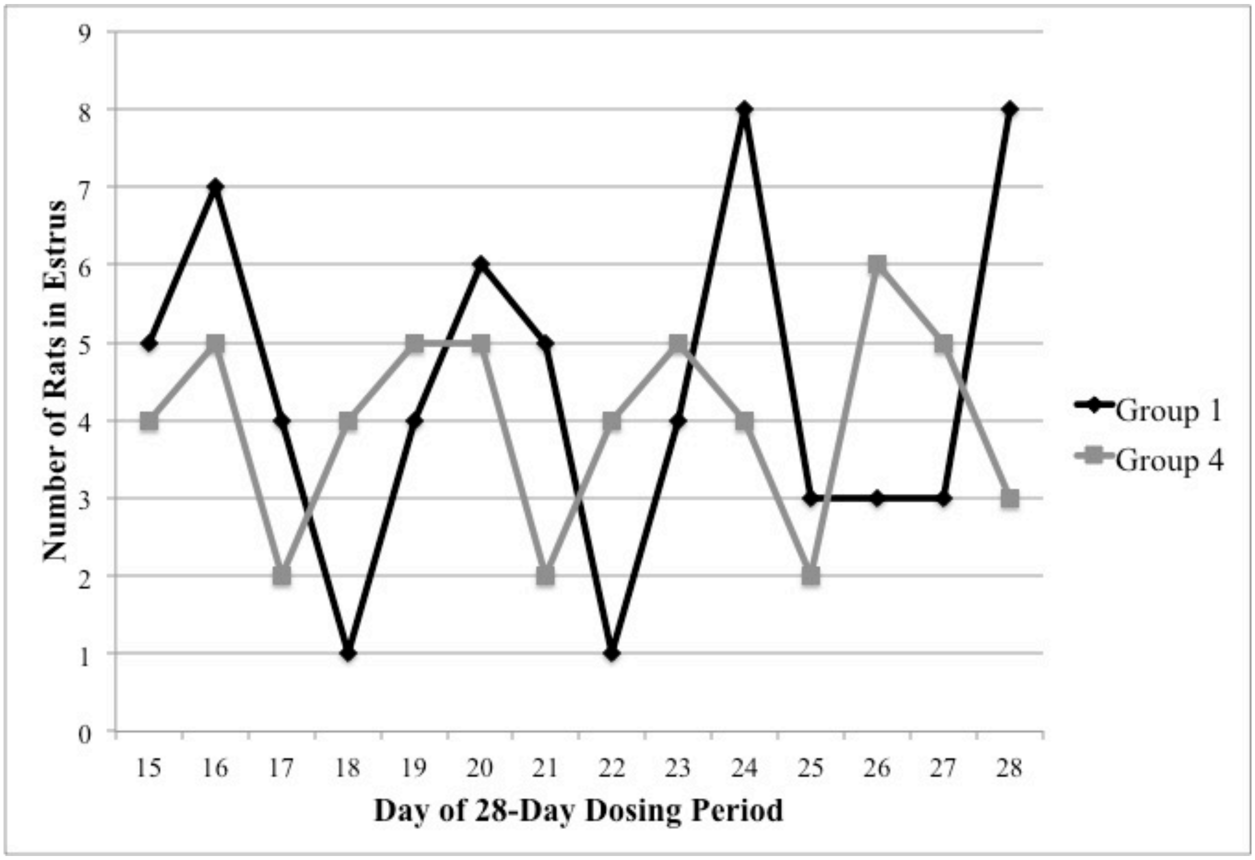

Supplement: Supplemental Material, DS1_IJT_10.1177_1091581818766318 - Safety Evaluation of Soy Leghemoglobin Protein Preparation Derived From Pichia pastoris, Intended for Use as a Flavor Catalyst in Plant-Based Meat [file DS1_IJT_10.1177_1091581818766318.pdf]
